# Base excision repair system targeting DNA adducts of trioxacarcin/LL-D49194 antibiotics for self-resistance

**DOI:** 10.1093/nar/gkac085

**Published:** 2022-02-22

**Authors:** Xiaorong Chen, Noah P Bradley, Wei Lu, Katherine L Wahl, Mei Zhang, Hua Yuan, Xian-Feng Hou, Brandt F Eichman, Gong-Li Tang

**Affiliations:** School of Chemistry and Materials Science, Hangzhou Institute for Advanced Study, University of Chinese Academy of Sciences, 1 Sub-lane Xiangshan, Hangzhou 310024, China; State Key Laboratory of Bioorganic and Natural Products Chemistry, Center for Excellence in Molecular Synthesis, Shanghai Institute of Organic Chemistry, University of Chinese Academy of Sciences, Chinese Academy of Sciences, Shanghai 200032, China; Department of Biological Sciences, Vanderbilt University, Nashville, TN 37232, USA; State Key Laboratory of Bioorganic and Natural Products Chemistry, Center for Excellence in Molecular Synthesis, Shanghai Institute of Organic Chemistry, University of Chinese Academy of Sciences, Chinese Academy of Sciences, Shanghai 200032, China; Department of Biological Sciences, Vanderbilt University, Nashville, TN 37232, USA; State Key Laboratory of Bioorganic and Natural Products Chemistry, Center for Excellence in Molecular Synthesis, Shanghai Institute of Organic Chemistry, University of Chinese Academy of Sciences, Chinese Academy of Sciences, Shanghai 200032, China; State Key Laboratory of Bioorganic and Natural Products Chemistry, Center for Excellence in Molecular Synthesis, Shanghai Institute of Organic Chemistry, University of Chinese Academy of Sciences, Chinese Academy of Sciences, Shanghai 200032, China; State Key Laboratory of Bioorganic and Natural Products Chemistry, Center for Excellence in Molecular Synthesis, Shanghai Institute of Organic Chemistry, University of Chinese Academy of Sciences, Chinese Academy of Sciences, Shanghai 200032, China; Department of Biological Sciences, Vanderbilt University, Nashville, TN 37232, USA; Department of Biochemistry, Vanderbilt University, Nashville, TN 37232, USA; School of Chemistry and Materials Science, Hangzhou Institute for Advanced Study, University of Chinese Academy of Sciences, 1 Sub-lane Xiangshan, Hangzhou 310024, China; State Key Laboratory of Bioorganic and Natural Products Chemistry, Center for Excellence in Molecular Synthesis, Shanghai Institute of Organic Chemistry, University of Chinese Academy of Sciences, Chinese Academy of Sciences, Shanghai 200032, China

## Abstract

Two families of DNA glycosylases (YtkR2/AlkD, AlkZ/YcaQ) have been found to remove bulky and crosslinking DNA adducts produced by bacterial natural products. Whether DNA glycosylases eliminate other types of damage formed by structurally diverse antibiotics is unknown. Here, we identify four DNA glycosylases—TxnU2, TxnU4, LldU1 and LldU5—important for biosynthesis of the aromatic polyketide antibiotics trioxacarcin A (TXNA) and LL-D49194 (LLD), and show that the enzymes provide self-resistance to the producing strains by excising the intercalated guanine adducts of TXNA and LLD. These enzymes are highly specific for TXNA/LLD-DNA lesions and have no activity toward other, less stable alkylguanines as previously described for YtkR2/AlkD and AlkZ/YcaQ. Similarly, TXNA-DNA adducts are not excised by other alkylpurine DNA glycosylases. TxnU4 and LldU1 possess unique active site motifs that provide an explanation for their tight substrate specificity. Moreover, we show that abasic (AP) sites generated from TxnU4 excision of intercalated TXNA-DNA adducts are incised by AP endonuclease less efficiently than those formed by 7mG excision. This work characterizes a distinct class of DNA glycosylase acting on intercalated DNA adducts and furthers our understanding of specific DNA repair self-resistance activities within antibiotic producers of structurally diverse, highly functionalized DNA damaging agents.

## INTRODUCTION

Genome stability and integrity are continually challenged by both intrinsic and extrinsic genotoxic agents that generate a diversity of DNA damage through oxidation, alkylation, or hydrolytic deamination (1). Among the most common forms of damage are those derived from alkylating agents, which can potentially modify any of the heteroatoms in duplex DNA. Different sites are alkylated depending on the nature of the DNA-alkylating agents. The resulting DNA damage—including single or double strand breaks, inter- or intra-strand crosslinks, base detachment and base modification—interferes with normal cellular processes, causing DNA mutations, chromosomal rearrangements and instability, which can contribute to heritable diseases and even cell death (2,3). Due to their cytotoxicity, DNA damaging agents often possess certain antimicrobial or antitumor activities, and some of them are used extensively as drugs in cancer treatment (4–8).

In the cell, DNA damage is repaired by several highly conserved pathways (2). Alkylated DNA is eliminated from the genome predominantly by direct reversal, base excision repair (BER), or nucleotide excision repair (NER) pathways (9–13). Direct reversal enzymes (e.g. alkylguanine DNA alkyltransferases and AlkB-family dioxygenases) extract alkyl substituents from the nucleobase to leave the nucleotide and DNA backbone intact, and can remove not only small base modifications, but also inter-strand DNA crosslinks and bulky exocyclic DNA adducts (14–16). BER also removes mainly small but also some bulky and crosslinked adducts (17–19), and is initiated by DNA glycosylases that liberate a single modified nucleobase from the DNA backbone through hydrolysis of the N-glycosidic bond (19–23). This reaction forms an apurinic/apyrimidinic (AP, or abasic) site that is then incised by an AP endonuclease (e.g. Exonuclease III (*Xth*) or Endonuclease IV (EndoIV, *Nfo*) in bacteria), generating a gap in the DNA backbone. In contrast, the NER pathway removes bulky or duplex-distorting lesions by endonuclease-catalyzed incisions that isolate a lesion-containing DNA oligonucleotide (24,25). DNA gaps generated in BER and NER are processed, filled, and sealed by the action of a DNA polymerase and DNA ligase.

**Figure 1.**
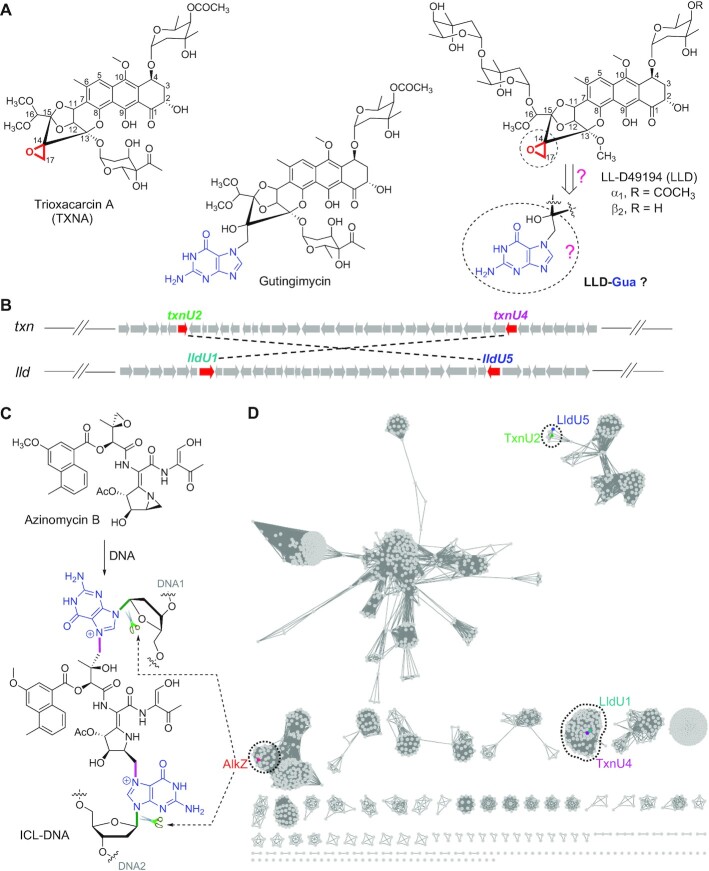
Structures of TXNs family compounds and genomic analysis of self-resistance determinants TxnU2/U4 and LldU1/U5. (**A**) Structures of trioxacarcin A (TXNA), gutingimycin, LL-D49194 (LLD) and LLD-guanine (LLD-Gua). Reactive epoxide moieties are highlighted in red. Guanine nucleobases are highlighted in blue. (**B**) Biosynthetic gene clusters (BGC) containing genes encoding HTH_42 superfamily proteins TxnU2/U4 and LldU1/U5. The two genes connected with dashed lines encode homologous proteins; TxnU2 shares 83% amino acid sequence identity and 90% similarity with LldU5, and TxnU4 shares 71% amino acid sequence identity and 82% similarity with LldU1. (**C**) Base excision of AZB-ICL-DNA by AlkZ. (**D**) Sequence similarity network (SSN) analysis of homologous proteins TxnU2/U4 and LldU1/U5. The SSN was constructed by the online Enzyme Function Initiative-Enzyme Similarity Tool with an alignment score threshold of 110. The proteins TxnU2/U4, LldU1/U5 and AlkZ were located in three different clades.

Recent studies of self-resistance mechanisms against genotoxic natural products revealed that several unrelated glycosylases participate in removing bulky adducts (26,27). Among them, the DNA glycosylase AlkZ, derived from *Streptomyces sahachiroi* and which resides within the biosynthetic gene cluster (BGC) of the natural product azinomycin B (AZB), repairs interstrand crosslink (ICL) damage generated by AZB (27–29). AZB is a bifunctional alkylating agent that forms ICLs in the major groove by linking the N7 nitrogens of purines in the duplex DNA sequence 5′-d(PuNPy)-3′ (30). AlkZ unhooks AZB-ICLs by cleaving the *N*-glycosidic bonds of both modified nucleotides, resulting in AP sites that can be processed by the BER pathway (Figure 1C) (19,27). The crystal structure revealed that AlkZ adopts a C-shaped structure in which the concave channel contains a QΦQ motif essential for catalytic activity and a β-hairpin predicted to contact the lesion in the minor groove (28). AlkZ belongs to the uncharacterized HTH_42 superfamily of proteins widespread in antibiotic producers and pathogenic bacteria (27). To date, the only other bacterial DNA glycosylase characterized as an ICL glycosylase is another HTH_42 protein, *Escherichia coli* YcaQ, which has a relaxed specificity relative to *S. sahachiroi* AlkZ and can cleave *N*7-linked nitrogen mustard (NM) ICLs and *N*7-methyl-2′-deoxyguanosine (7mG) monoadducts (29).

Trioxacarcins (TXNs) are densely oxygenated, polycyclic aromatic, and structurally complex natural products with potent cytotoxicity (Figure 1A) (31–34). Trioxacarcin A (TXNA) and LL-D49194 (LLD), two of the most representative compounds in the TXN family, intercalate the base pairs of DNA and have reactive epoxide moieties that covalently alkylate the N7 of guanine in d(GT) dinucleotides, forming stable DNA lesions that impair normal cellular processes (35,36). Consequently, TXNA and LLD exhibit remarkable antimalarial, antibacterial and antitumor activity (31,33,34). The TXNA analog gutingimycin (Figure 1A), which contains a TXN skeleton and a guanine (Gua) group, has been isolated from the fermentation broth of a marine *Streptomycete* (37). Given that TXNA and LLD are alkylating agents that selectively modify deoxyguanosine (G) to form DNA adducts, we speculated that the biosynthetic pathways of the two natural products should contain DNA glycosylases responsible for cleaving TXNA/LLD-DNA, in which gutingimycin and LLD-Gua are the resulting products (Figure 1A). Therefore, we became interested in the DNA damage repair mechanism targeting TXNs family of DNA alkylating agents.

Herein, we report four DNA glycosylases identified from the TXNs BGC, in which TxnU2/U4 are derived from the TXN BGC (*txn*, GenBank accession number KP410250) and LldU1/U5 originate from the LLD BGC (*lld*, GenBank accession number MK501817) (Figure 1B). TxnU2/4 and LldU1/5 belong to the HTH_42 superfamily and are monofunctional DNA glycosylases that excise TXNA- and LLD-DNA adducts, in which TxnU4 and LldU1 play the major roles in toxin resistance. Interestingly, TxnU4 and LldU1 cannot excise *N*7-methyl or crosslinked G adducts like their homologs AlkZ and YcaQ (28,29), nor can TXNA-DNA lesions be excised by any other alkylpurine DNA glycosylase. Moreover, relative to AlkZ, TxnU4 and LldU1 have a unique catalytic motif that process TXNA- and LLD-DNA lesions differently and that may explain the redundancy for two paralogs in each *txn* and *lld* biosynthetic gene cluster. We also show that AP sites derived from TXNA-DNA excision are processed less efficiently than those generated from 7mG depurination, suggesting that the product of TXNA-DNA excision requires a specialized mechanism for repair.

## MATERIALS AND METHODS

### Reagents

Expression vector pBG102 (Supplementary Table S1) was obtained from the Vanderbilt University Center for Structural Biology. DNA oligonucleotides (Supplementary Table S2) were purchased from Integrated DNA Technologies. AlkA, AlkC, AlkD, AlkZ and YcaQ were purified as previously described (28,29,38–40). *E. coli* EndoIV was purchased from New England BioLabs. Unless otherwise noted, all chemicals were purchased from Sigma-Aldrich. TXNA and LLD were isolated from *S. bottropensis* NRRL 12051 and *S. vinaceusdrappus* NRRL 15735, respectively, as described below.

### Sequence similarity network (SSN) analysis

The 15,119 homologous proteins of AlkZ were obtained from the InterPro website (41) (http://www.ebi.ac.uk/interpro/search/sequence-search) by using AlkZ as the query. Sequences were then clustered by CD-HIT Suite (42) on the website (http://weizhong-lab.ucsd.edu/cdhit_suite/cgi-bin/index.cgi?cmd=cd-hit) with 53% sequence identity threshold. The representatives of the resulting clusters and TxnU2, TxnU4, LldU1, LldU5, AlkZ were used for construction of SSN by the online Enzyme Function Initiative-Enzyme Similarity Tool (43) with an alignment score threshold of 110. Cytoscape software was used to view the sequence similarity networks.

### Fermentation and isolation of TXNA and LLD

For TXNA production, *Streptomyces bottropensis* NRRL 12051 and its relative mutant strains were cultivated as previously reported (44). After fermentation in SYG medium (soluble starch 60 g/l, glucose 10 g/l, yeast extract 10 g/l, NaCl 3 g/l, MgSO_4_•7H_2_O 1 g/l, KH_2_PO_4_ 1 g/l, CuSO_4_•5H_2_O 70 mg/l, FeSO_4_•7H_2_O 10 mg/l, MnCl_2_•4H_2_O 8 mg/l, ZnSO_4_•7H_2_O 2 mg/l, CoCl_2_•7H_2_O 6 μg/l, HP20 30 g/l) for 5 days, the TXNA was isolated and detected as described (45). The fermentation and isolation of LLD was similar to TXNA (46). *S. vinaceusdrappus* NRRL 15735 and those mutants were cultivated in SYG medium for 10 days, and then isolated and detected by HPLC. HPLC analysis was performed on an Acclaim 120 C18 column (5 μm, 4.6 × 250 mm) at a flow rate of 1.0 ml/min and a linear gradient program: 0–5 min, 10% phase B (0.1% formic acid in CH_3_CN); 5–24 min, solvent B gradient from 10 to 90% followed with 90% B at 24–26 min; 26–27 min, gradient from 90 to 10% B; 27–31 min, constant 10% B. Phase A is 0.1% formic acid in H_2_O. TXNA/LLD-related compounds were determined by measuring UV absorbance at 400 nm using an Agilent 1200 series system (45,46). LC–MS was carried out on a ThermoFisher LTQ XL under the same conditions.

### Cellular TXNA and LLD self-resistance assays

#### Zone of inhibition assays in streptomyces

The inhibition zones of *Streptomyces* were performed by a disc diffusion assay. Specifically, filter paper discs spotted with different concentrations of TXNA or LLD were laid on the MS plate (20 g/l soybean meal, 20 g/l mannitol, 20 g/l agar, pH 7.2), which were pre-inoculated with wild-type strains *S. bottropensis* NRRL 12051 (*txn*WT), *S. vinaceusdrappus* NRRL 15735 (*lld*WT), the gene mutant strains, *ΔtxnU2*, *ΔtxnU4*, *ΔlldU1*, *ΔlldU5* or heterologous expression strains *S. lividans*::pSET152, *S. lividans*::*txnU2*, *S. lividans*::*txnU4*, *S. lividans*::*lldU1*, *S. lividans*::*lldU5* (Supplementary Table S1). After incubation at 30°C for 36 h, resistance levels to TXNA or LLD were determined by the zone of inhibition.

#### Heterologous survival assays in E. coli

*E. coli* BL21 cells transformed with protein overexpression plasmid *txnU2*-pET28a, *txnU4*-pET28a, *lldU1*-pET28a, *lldU5*-pET28a or empty vector pET28a alone were grown overnight at 37°C in LB medium containing 50 μg/ml kanamycin (Kan). The overnight cultures were then transferred to fresh LB medium supplemented with 50 μg/ml Kan and incubated at 30°C. When the OD_600_ reached 0.6, 0.1 mM isopropyl β-d-1-thiogalactopyranoside (IPTG) was added to induce protein expression. After growing at 16°C for 2 h, cells were diluted to 0.01 OD_600_ in 2 ml fresh LB supplemented with Kan and IPTG. The dilutions were treated with various concentrations of TXNA for 12 h at 30°C and cell density was measured by OD_600_. The surviving fraction (%) was calculated as (OD_600_(treated)/OD_600_(untreated)) × 100. The data were fit by non-linear regression and plotted using GraphPad 8.0 software.

### TxnU2/4 and LldU1/5 purification

The *lldU1/5* (GenBank accession numbers QDQ37873 and QDQ37896) and *txnU2/4* (GenBank accession numbers AKT74276 and AKT74302) genes were synthesized by GenScript and cloned into pBG102 (Vanderbilt Center for Structural Biology). N-terminal His_6_-SUMO proteins were overexpressed in *E. coli* Tuner (DE3) cells at 16°C for 18 h in LB medium supplemented with 30 μg/ml kanamycin and 50 μM isopropyl β-d-1-thiogalactopyranoside (IPTG). Cells were lysed with sonication and cell debris removed by centrifugation at 45 000 × g at 4°C for 30 min. Clarified lysate was passed over Ni-NTA agarose equilibrated in buffer A (50 mM Tris•HCl pH 8.5, 500 mM NaCl, 20 mM imidazole, and 10% (vol/vol) glycerol) and protein eluted in 250 mM imidazole/buffer A. Protein fractions were pooled and supplemented with 0.1 mM EDTA, 1 mM tris(2-carboxyethyl)phosphine (TCEP), and 1 mM dithiothreitol (DTT) before incubation with 0.5 mg of Rhinovirus 3C (PreScission) protease and 0.5 mg of yeast ubiquitin-like-specific protease 1 (Ulp1) at 4°C overnight. Cleaved protein was diluted 10-fold in buffer B (50 mM Tris•HCl pH 8.5, 10% (vol/vol) glycerol, 0.1 mM TCEP, and 0.1 mM EDTA) and purified by heparin sepharose using a 0–1 M NaCl/buffer B linear gradient. Fractions were pooled and repassed over Ni-NTA agarose in buffer A, concentrated and filtered, and buffer exchanged into buffer C (20 mM Tris•HCl pH 8.5, 100 mM NaCl, 5% (vol/vol) glycerol, 0.1 mM TCEP, and 0.1 mM EDTA). Proteins were concentrated to 100 μM, flash-frozen in liquid nitrogen, and stored at −80°C. For purification of TxnU2 and LldU5, buffers A and B were supplemented with 0.02% NP-40 and buffer C was supplemented with 0.01% NP-40. Proteins used in HPLC analysis did not contain NP-40. *LldU1/5* and *txnU2/4* mutants were generated using the Q5 Mutagenesis Kit (New England BioLabs). Mutant proteins were overexpressed and purified the same as WT.

### Preparation of DNA substrates

The TXNA- and LLD-DNA substrates for HPLC analysis, which contained two lesions per duplex, were prepared by annealing the 8-bp self-complementary strand 5′-AACCGGTT-3′ (36), followed by incubation of 50 μM DNA with 100 μM TXNA or LLD in PBS buffer (pH 7.0) at 16°C for 2 h. TXNA- and LLD-DNA substrates used in gel-based assays contained a single TXNA-G or LLD-G adduct and a 5′-cyanine 5 (Cy5) label, and were prepared by annealing the strand containing the TXNA/LLD target sequence (*TXN/LLD Top*, Supplementary Table S2) to the complementary unlabeled oligo (*TXN/LLD Bottom*, Supplementary Table S2), followed by incubation of 100 μM DNA with 200 μM TXNA or LLD in 10% methanol and 20% DMSO at 4°C on ice in the dark for 36 h. Unreacted drug was removed using a G-25 spin column equilibrated in TE buffer (pH 8.0), and the DNA was stored at -80°C. DNA substrates containing a single *N*7-methyl-2′-deoxyguanosine (7mG) lesion and a 5′-Cy5-label on one strand were prepared as described previously using *7mG_Top* and *7mG_Bottom* oligonucleotides (Supplementary Table S2) (47). NM-ICLs containing both 6-carboxyfluorescein (FAM) and Cy5 labels were generated using *NM_Top* and *NM_Bottom* oligonucleotides (Supplementary Table S2) and purified as reported previously (29).

### Base excision assays

#### HPLC analysis

A 50 μl reaction containing 50 μM TXNA- or LLD-DNA, 20 μM protein, and buffer (100 mM Na_2_HPO_4_, 100 mM NaH_2_PO_4_, 500 mM NaCl, pH 7.0) was incubated at 16°C for 2 h. The reaction mixtures were quenched with 30 μl methanol and analyzed by LC–MS at 400 nm absorbance. TXNA-Gua (gutingimycin), [M + H]^+^ ion with *m/z* 1028.53; LLD-G, [M + H]^+^ ion with *m/z* 1102.43.

#### Denaturing page analysis

Glycosylase reactions were performed with 50 nM DNA in glycosylase buffer (50 mM HEPES pH 8.5, 100 mM KCl, 1 mM EDTA, and 10% (vol/vol) glycerol) at 25°C. Single-timepoint reactions shown in Figures 4–6 were performed with 1 μM enzyme for either 30 s, 30 min, or 96 h, as indicated in each figure legend. Single- and multiple-turnover kinetics reactions shown in Figure 4G were performed with 50 nM (single turnover) or 5 nM (multiple turnover) TxnU4 and 50 nM Cy5-labeled TXNA-DNA. Thermal depurination controls shown in Figure 5A were conducted at 95°C for 5 min. Enzyme and mock reactions involving TXNA, LLD and 7mG monoadducts were quenched by adding 1 μl of 1 M NaOH to a 4-μl reaction aliquot and heating at 70°C for 2 min. Samples were denatured by addition of 5 μl loading buffer containing 5 mM EDTA pH 8.0, 80% (wt/vol) formamide, and 1 mg/ml blue dextran, and incubating at 70°C for 5 min. Samples were electrophoresed on a 20% (wt/vol) acrylamide/8 M urea sequencing gel at 40 W for 1.5 h in 0.5 × TBE buffer (45 mM Tris, 45 mM borate and 1 mM EDTA pH 8.0). Gels were imaged on a Typhoon Trio variable mode imager (GE Healthcare) for Cy5 fluorescence (633 nm excitation, 670 nm emission), and bands were quantified with ImageQuant (GE Healthcare). Percent product was calculated as the percent of both β- and δ-elimination bands divided by the total intensity of substrate and β/δ-elimination bands. Unreacted DNA in LLD-DNA reactions was not included in the calculation of percent product. NM-ICLs reactions were performed the same as monoadducts, but were quenched and denatured at 55°C prior to electrophoresis. Gels were imaged for both FAM (488 nm excitation, 526 nm emission) and Cy5 fluorescence and artificially colored (FAM, green; Cy5, red) using Adobe Photoshop and overlaid using ImageJ software as previously described (29). All excision assays were performed in triplicate.

#### Spontaneous depurination

Non-enzymatic depurination of G, 7mG and TXNA-G were conducted at 37°C in glycosylase buffer using 50 nM DNA, with the same Cy5-oligodeoxynucleotides described above. The G-DNA oligo was the same as that used to make the TXNA-G oligo. Samples were quenched and products quantified the same as the enzymatic reactions described above.

#### EndoIV abasic site incision kinetics

AP-DNA substrates were generated by incubation of 5 nM YcaQ or TxnU4 with 50 nM Cy5-(TXNA/7mG)-DNA in glycosylase buffer for 2 h at 25°C. EndoIV incision reactions were performed by adding 6 μl of 83 nM EndoIV (17 nM final concentration) to a 24-μl glycosylase reaction aliquot and incubating at 37°C. Reactions were heated at 70°C for 5 min with 5 μl of formamide/blue dextran loading buffer and electrophoresed and imaged as above. Curve fitting was performed in Prism 9 using a single exponential one-phase association for 7mG-AP site incision and an exponential two-phase association for TXNA-G-AP sites.

## RESULTS

### Self-resistance determinants TxnU2/U4 and LldU1/U5 are closely related to TXNs production

**Figure 2.**
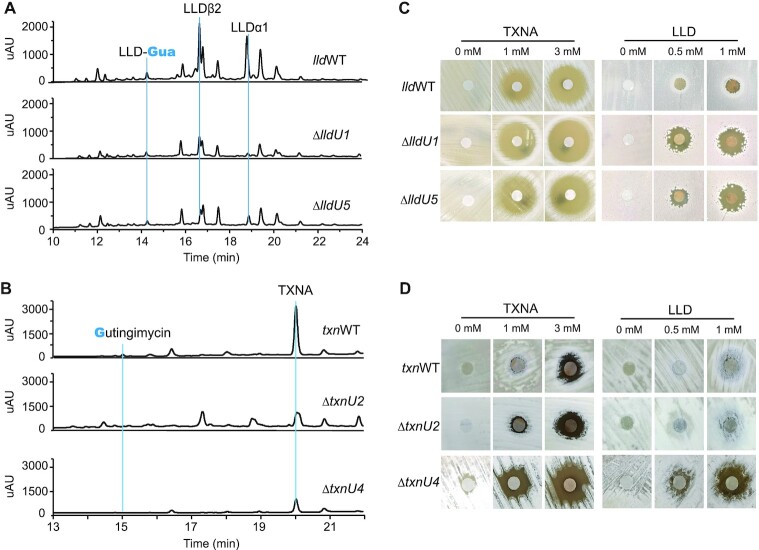
*In vivo* characterization of the self-resistance determinants related to LLD and TXNA. (**A**) LC–MS analysis of extracts from *S. bottropensis* NRRL 12051 wild-type (*txn*WT) and mutant strains, Δ*txnU2* and Δ*txnU4*, at 400 nm absorbance. (**B**) LC–MS profiles of extracts from *S. vinaceusdrappus* NRRL 15735 wild-type (*lld*WT) and mutant strains, Δ*lldU1* and Δ*lldU5*, at 400 nm absorbance. The effect of *txnU2*/*txnU4* (**C**) and *lldU1*/*lldU5* (**D**) deletion on cells challenged with increasing concentrations of TXNA (left) and LLD (right) was tested by a disc diffusion assay. Filter paper discs spotted with different concentrations of TXNA or LLD were laid on the MS plate pre-inoculated with wild type or mutant strains. After incubation at 30°C for 36 h, resistance levels to TXNA or LLD were determined by the zone of inhibition.

Previously, we identified the BGCs of TXNA (*txn*) and LLD (*lld*) and characterized their partial biosynthetic pathways including starter unit and tailoring steps (45,46,48–51), but the function of many of the proteins encoded in their BGCs are unknown. To study the repair mechanism of DNA damage arising from TXNs family of alkylating agents, we first investigated all proteins encoded within and adjacent to the TXNA and LLD BGCs (45,46). BLASTP analysis showed that TxnU2/U4 derived from *txn a*nd LldU1/U5 derived from *lld* belong to the HTH_42 superfamily and exhibit homology to the DNA glycosylase AlkZ with low sequence identity (26–33%) and similarity (39–46%) (Figure 1B and D). AlkZ is found within the AZB BGC and has been reported to be an essential resistance protein in AZB biosynthesis by unhooking AZB-ICLs, which would trigger the BER pathway (Figure 1C) (27). We therefore speculated that TxnU2/U4 and LldU1/U5 could confer resistance to TXNA and LLD for self-protection in the producer. To understand the function of these four proteins, the genes *txnU2*/*U4* from the TXNA producer *S. bottropensis* NRRL 12051 and *lldU1*/*U4* from the LLD producer *S. vinaceusdrappus* NRRL 15735 were deleted (Supplementary Figure S1), and the yield of compounds in these resulting mutants and wild-type (WT) strains were determined by LC–MS. Compared to the WT strain, the production of TXNA in gene deletion mutant strains Δ*txnU2* and Δ*txnU4* was remarkably reduced 72% and 82%, respectively, and the yield of LLD in Δ*lldU1* and Δ*lldU5* was also decreased 85% and 80%, respectively, suggesting the genes *txnU2*/*txnU4* and *lldU1*/*lldU5* are involved in compound biosynthesis and are closely related to the efficiency of TXNA and LLD production (Figure 2A and B).

**Figure 3.**
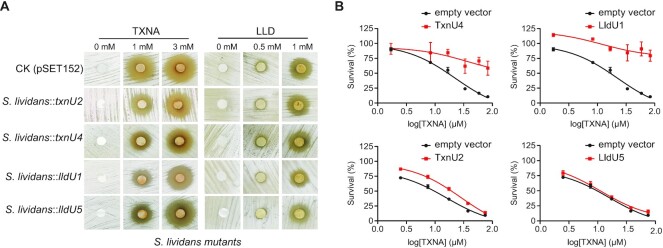
Overexpression of TxnU2/TxnU4 and LldU1/lldU5 confer resistance to heterologous hosts against TXNA and LLD. (**A**) Disc diffusion test assay to determine the antibiotic sensitivity of heterologous expression strains *S. lividans*::pSET152, *S. lividans*::*txnU2*, *S. lividans*::*txnU4*, *S. lividans*::*lldU1* and *S. lividans*::*lldU5* to TXNA (left) and LLD (right). (**B**) TXNA inhibition of *E. coli* BL21 cells transformed with protein overexpression plasmid *txnU2*-pET28a, *txnU4*-pET28a, *lldU1*-pET28a, *lldU5*-pET28a or empty vector pET28a alone. Data are mean ± SD (*n* = 3).

To follow up this finding and further identify the *in vivo* function of the four proteins, the effects of *txnU2*/*txnU4*and *lldU1*/*lldU5* deletion and overexpression on cells challenged with TXNs were tested. Disc diffusion tests indicated that gene deletion mutants Δ*txnU4*, Δ*lldU1* and Δ*lldU5* exhibited notable sensitivity to both TXNA and LLD, but mutant Δ*txnU2* was no more sensitive to either TXNA or LLD than the WT strain (Figure 2C and D). Overexpression of *txnU2*/*txnU4* and *lldU1*/*lldU5* in *S. lividans* 1326, a TXNs-sensitive strain, increased cellular viability towards both TXNA and LLD (Figure 3A). Moreover, consistent with the growth viability in *Streptomyces*, the survival ratio of *E. coli* BL21 that overexpressed *txnU4* or *lldU1* against TXNA was significantly higher than control cells, while *txnU2* overexpression was weakly protective, and there was no effect for *lldU5* overexpression (Figure 3B). Together, these results show that TxnU2/U4 and LldU1/U5 are self-resistance determinants in TXNA and LLD producers, and among them TxnU4 and LldU1 display the major roles.

### TxnU2/U4 and LldU1/U5 are DNA glycosylases that excise TXNA- and LLD-DNA adducts

**Figure 4.**
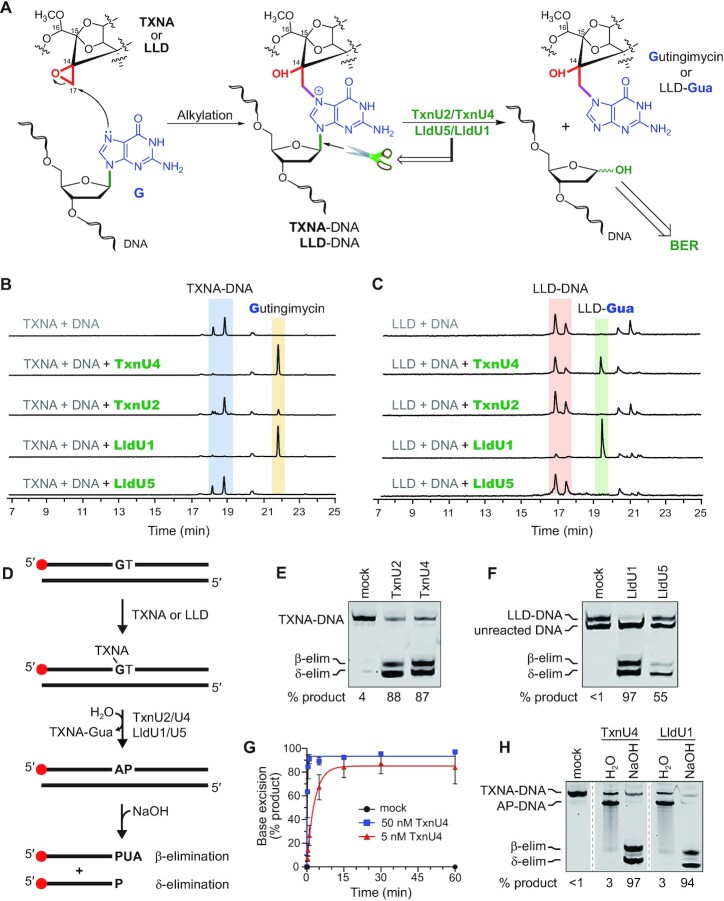
TxnU2/4 and LldU1/5 are monofunctional DNA glycosylases that excise TXNA- and LLD-DNA adducts. (**A**) Chemical reaction between the epoxide moiety of TXNA or LLD and N7 of G in DNA. DNA glycosylases catalyze the hydrolysis of the N-glycosidic bond to liberate the alkylguanine adduct, generating an AP site in the DNA. (B, C) LC–MS analysis of the cleavage products of TxnU2, TxnU4, LldU1 and LldU5 reaction with TXNA-DNA (**B**) and LLD-DNA (**C**). An 8-bp oligodeoxynucleotide duplex d(AACCGGTT) was pre-incubated with TXNA or LLD at 16°C for 2 h, followed by treatment with enzymes TxnU2, TxnU4, LldU1 and LldU5 for 2 h. The reaction mixtures were analyzed by LC–MS at 400 nm absorbance. (**D**) Schematic of the base excision assay performed in panels E-H. DNA containing a centrally located GT dinucleotide and a 5′-Cy5-label (red circle) is incubated with TXNA or LLD to form the substrate. Incubation with TxnU2/U4 or LldU1/U5 generates an AP site, which is cleaved with hydroxide to generate β- and δ-elimination products. PUA, 3′-phospho-α,β-unsaturated aldehyde; P, 3′-phosphate. (E, F) Denaturing PAGE of TXNA-DNA (**E**) and LLD-DNA (**F**) reactions after treatment with enzyme or buffer (mock) for 30 min. Formation of the LLD-DNA substrate only went to ∼50% completion, with unreacted DNA migrating faster on the gel. Substrate and product DNA migrate as expected for their sizes, as judged by their relative position to bromophenol blue and xylene cyanol tracking dyes (Supplementary Figure S5C) (66). (**G**) Single- (blue) and multiple-turnover (red) excision kinetics of TxnU4 against TXNA-DNA. 50 nM TXNA-DNA was incubated with buffer (mock), 50 nM TxnU4 (1:1 protein:DNA), or 5 nM TxnU4 (1:10 protein:DNA). Data are mean ± SD (*n* = 3). A representative gel from which the data were quantified is shown in Supplementary Figure S5D. (**H**) Denaturing PAGE of TXNA-DNA adducts after 30-min incubation with TxnU4 or LldU1, followed by work-up with either H_2_O or NaOH.

To determine if TxnU2, TxnU4, LldU1 and LldU5 are DNA glycosylases capable of excising TXNA- and LLD-Gua adducts from DNA, an 8-bp oligodeoxynucleotide duplex d(AACCGGTT) designed based on a previous report was treated with either TXNA or LLD and then incubated with TxnU2, TxnU4, LldU1, or LldU5 (Figure 4A) (36,52). The reaction products were detected by LC–MS at 271 and 400 nm (Figure 4B, C, Supplementary Figure S2). After treatment with TXNA, two new peaks appeared at 18.2 and 18.9 minutes. The *m/z* of the two peaks were 1644, which was consistent with that of the [M + 2H]^2+^ ion of the monoalkylated adduct generated by covalent binding of one molecule TXNA to either G within the duplex d(AACCGGTT) (Supplementary Figure S2A). Given the previous sequence selectivity studies showing that TXNA reacts preferentially with the DNA sequence 5′-GT (36,52), we supposed that the product with the later retention time (18.9 min) and larger peak area is 5′-AACCG(TXNA-G)TT-3′, and the other peak at 18.2 minutes is 5′-AACC(TXNA-G)GTT-3′. As TxnU2 or TxnU4 was added, the amount of the two adducts decreased, and a new peak with *m/z* 1028 appeared, which was supposedly the excision product of TxnU2 and TxnU4 (Figure 4B). The molecular weight of the product is equal to that of gutingimycin, which contains a TXN skeleton and a Gua nucleobase. In addition, the molecular formula C_47_H_57_O_21_N_5_ determined by HRESIMS ([M + H]^+^*m/z* 1028.53) and the fragments detected by tandem-MS were consistent with gutingimycin (Supplementary Figure S3A), confirming that TxnU2 and TxnU4 are able to catalyze excision of TXNA-Gua adducts from DNA. An extended time course indicated that TxnU4 preferentially cleaved the 5′-AACCG(TXNA-G)TT-3′ among the two alkylated products (Supplementary Figure S4). Likewise, under the same experimental condition, the two alkylation products arising from LLD were excised by LldU1, forming a new compound with *m/z* 1102 in the mass spectra, whereas LldU5 showed no activity (Figure 4C, Supplementary Figure S2B). HRESIMS data ([M + H]^+^*m/z* 1102.43, calculated for C_51_H_68_O_22_N_5_) and tandem-MS analysis indicated that the excision product is LLD-Gua (Supplementary Figure S3B), suggesting LldU1 is capable of excising LLD-G adducts from DNA.

For further confirmation, an *in vitro* gel-based assay was performed to quantify the β- and δ-elimination products generated by alkaline hydrolysis of the AP site product of base excision (Figure 4D) (28). We verified that the amount of product observed in this assay was not influenced by the use of NaOH to cleave glycosylase generated AP sites, as similar results were obtained with piperidine (Supplementary Figure S5A,B). Purified enzymes were incubated with either TXNA- or LLD-DNA substrates for 30 min under single turnover conditions. We found that all four enzymes produced a significant amount of product as compared to a no-enzyme control (Figure 4E and F). The weaker activity of LldU5 relative to the other three enzymes (Figure 4F) is likely the result of poor protein solubility observed during expression and purification. Single-turnover kinetic analysis showed that TXNA-Gua excision by TxnU4 (*k*_st_ = 4.6 min^–1^) is approximately 4 times faster than *S. sahachiroi* AlkZ and *E. coli* YcaQ activity toward AZB-ICL (*k*_st_ = 1.2 min^–1^) and NM-ICL (*k*_st_ = 1.1 min^–1^) substrates, respectively (29) (Figure 4G, Supplementary Figure S5D). The enzyme also efficiently turns over (*k*_mt_ = 0.3 min^–1^) and shows no observable product inhibition, as evidenced by multiple-turnover kinetics (Figure 4G, Supplementary Figure S5D). Thus, these enzymes excise TXNs lesions rapidly and efficiently relative to their distant orthologs. Moreover, the *in vitro* excision activities of TxnU2/U4 and LldU1/U5 were further confirmed by the detection of excision products in gene deletion mutant strains (Figure 2A, B). Compared to the WT strain, the production of LLD-Gua in gene deletion mutant strains Δ*lldU1* and Δ*lldU5* was respectively reduced 43% and 30%, and the yield of gutingimycin in Δ*txnU2* and Δ*txnU4* was also respectively decreased 95% and 99%, suggesting the glycosylases TxnU2/U4 and LldU1/U5 are functional *in vivo*.

Monofunctional glycosylases catalyze only hydrolysis of the N-glycosidic bond, whereas bifunctional glycosylases also nick the backbone to generate β- and δ-elimination products. Based on our previous functional analysis of the homolog AlkZ, we hypothesized that TxnU and LldU enzymes were monofunctional. Indeed, similar to AlkZ, NaOH was required to nick the AP-DNA product formed by TxnU4 and LldU1 (Figure 4H). Treating the reacted TXNs-DNA with water preserved the AP site, while treatment with hydroxide cleaved the AP site to generate β- and δ- elimination products. These results indicate that the TxnU and LldU enzymes are monofunctional glycosylases and do not contain intrinsic DNA lyase activity.

### TxnU4 and LldU1 remove TXNs-guanine adducts with a similar but distinct catalytic motif relative to AlkZ

The active sites of all monofunctional DNA glycosylases contain catalytic carboxyl (Asp, Glu) or carboxamide (Asn, Gln) residues that promote base excision by electrostatically stabilizing the positive charge that develops on the deoxyribose as the glycosidic bond is broken, and by deprotonating or positioning a water molecule for nucleophilic attack of the anomeric C1′ carbon (19–23). We previously showed that the TxnU/LldU homolog AlkZ contains a catalytic QΦQ motif (Φ is a small aliphatic residue) (Supplementary Figure S6), and that mutation of either flanking glutamine abrogates base excision of monoadducts and severely reduces ICL unhooking activities (28,29). Based on a rigid-body docking model of AlkZ in complex with AZB-DNA (28), the C-terminal glutamine side chain is likely within proximity to the lesion deoxyribose to position a catalytic water molecule (Supplementary Figure S7). Although the N-terminal glutamine is more recessed and contacts the DNA backbone of a neighboring nucleotide, a slight rotation of the DNA around the helical axis in our docking model would position this residue for catalysis on the adducted nucleotide, and thus either residue theoretically can play a catalytic role in base excision.

**Figure 5.**
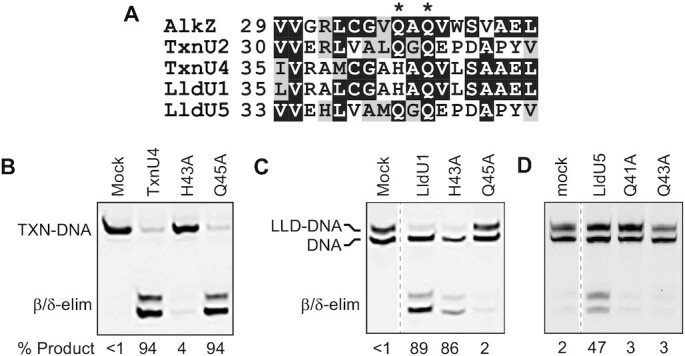
Mutational analysis of excision activity. (**A**) Sequence alignment of the catalytic residues in *S. sahachiroi* AlkZ and TxnU2/U4 and LldU1/U5. Denaturing PAGE of TxnU4/TXNA-DNA (**B**) and LldU1/LLD-DNA (**C**) Single-turnover reactions containing 1 μM protein and 50 nM DNA. WT and mutant proteins were incubated with substrates for 30 s. (**D**) Single turnover reactions between LldU5 enzymes and LLD-DNA were carried out for 96 h.

Like AlkZ, TxnU2 and LldU5 contain a QΦQ motif, whereas TxnU4 and LldU1 contain a histidine residue (H43) in the first position (Figure 5A). Both QΦQ and HΦQ motifs are predicted to reside in the same location as those observed in AlkZ (Supplementary Figures S6 and S7), and the His imidazole should be able to perform the same catalytic function as described above for carboxylate and carboxamide side chains. We examined the functional role of the HΦQ motifs in TxnU4 and LldU1 by purifying H43A and Q45A mutants and measuring TXNA-DNA and LLD-DNA excision activity. Wild-type TxnU4 removed 94% of the TXNA-DNA adduct after 30 seconds. At this same short time point, the TxnU4 H43A mutant showed no activity, whereas substitution of Gln45 with alanine had no effect on TxnU4 activity (Figure 5B). Interestingly, we found the exact opposite effect of H43 and Q45 residues in LldU1 tested against an LLD-DNA substrate; LldU1 H43A had no effect compared to wild-type, whereas LldU1 Q45A showed no activity (Figure 5C). We also tested the activity of the QΦQ motif in LldU5; alanine substitution of either glutamine abrogated activity compared to the wild-type enzyme (Figure 5D), similar to that shown for AlkZ (28). We were unable to test the activity of TxnU2 mutants because the proteins were unstable and not amenable to purification. These results indicate that the *in vitro* activity we observe from purified protein is not the result of a contaminating activity in our protein preparations, and suggest that either the histidine or glutamine residues within TxnU4 and LldU1 HΦQ motifs are catalytic, and that they engage TXNA-G and LLG-G lesions differently (Supplementary Figure S7D).

### TXNs form stable DNA adducts that are specifically excised by TxnU and LldU glycosylases

**Figure 6.**
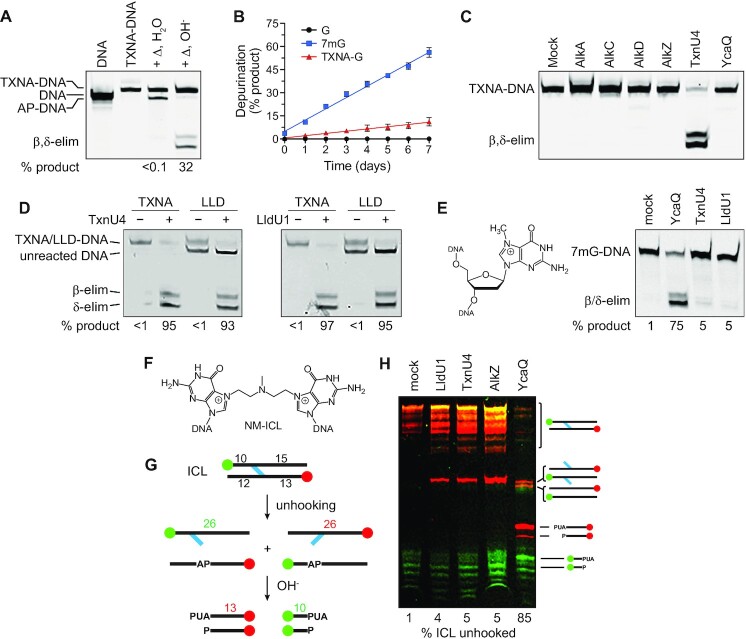
Substrate specificity analysis of TxnU2/TxnU4 and LldU1/5. (**A, B**) TXNA forms stable DNA adducts. Denaturing PAGE of TXNA-DNA adducts after thermal depurination. TXNA-DNA was heated (Δ) to 95°C for 5 min, followed by treatment with either water or NaOH. (B) Kinetics of spontaneous depurination of TNXA in DNA as compared with 7mG or unmodified G. Data are mean ± SD (*n* = 3). Half-lives derived from linear regression of the data are 6.1 ± 0.3 days (7mG) and 33.9 ± 8.1 days (TXNA). A representative gel from which this data was quantified is shown in Supplementary Figure S5E. (**C**) Denaturing PAGE of TXNA-DNA adducts after 1-h incubation with either buffer (mock) or bacterial alkyl-DNA glycosylases. (**D**) TxnU4 can excise LLD-DNA and LldU1 can excise TXN-G-DNA adducts. (**E**) Denaturing PAGE of 30-min reaction products of *E. coli* YcaQ and *Streptomyces* TxnU4 and LldU1 with 7mG-DNA. (**F**) Structure of nitrogen mustard (NM)-ICL produced by reaction of mechlorethamine with guanines on opposite DNA strands. (**G**) Schematic of ICL unhooking reactions. Strands are 5′-labeled with either FAM (green) or Cy5 (red). Unhooking by a glycosylase produces single stands containing either monoadducts or AP-sites, the latter which are susceptible to nicking by hydroxide. (**H**) Denaturing PAGE of NM-ICL unhooking reactions after treatment with buffer (mock) or enzyme for 30 min, followed by alkaline hydrolysis. The percent of β/δ-elimination products is quantified below the gel. Each image is an overlay of false-colored FAM (green) and Cy5 (red) fluorescence scans of the gels, in which yellow depicts coincident red and green intensity.

*N*7-alkyl-2′-deoxyguanosine adducts (e.g. 7mG) are generally thermally unstable and prone to depurination (53). We therefore explored the stability of TXNs-DNA adducts. Heating the TXNA-DNA to 95°C for 5 min, followed by either water or hydroxide workup, led to depurination of only 32% of the adduct (Figure 6A). In contrast, our previous studies show 90% depurination of *N*7-linked NM- and AZB-ICLs under the same conditions (29), suggesting that TXNA-DNA adducts are more stable than other *N*7-alkyl lesions. To test this, we directly compared the stabilities of TXNA-DNA and 7mG-DNA adducts by monitoring their spontaneous depurination rates at 37°C over a period of 7 days. We found that the TXNA-G N-glycosidic bond is at least 5 times more stable than that of 7mG (Figure 6B, Supplementary Figure S6). Thus, relative to 7mG, TXNA adducts are more resistant to spontaneous depurination, which may be an important property for TXNs toxicity.

We next tested the ability of other bacterial alkylpurine DNA glycosylases to liberate gutingimycin from DNA. These glycosylases, which include *E. coli* AlkA and YcaQ, *Bacillus cereus* AlkC and AlkD, and *S. sahachiroi* AlkZ, have widely varying substrate specificities in addition to their ability to excise 7mG (23,28,29,40,54–56). Under the experimental conditions tested, we were unable to detect TXNA excision products from any of these glycosylases (Figure 6C), indicating that recognition of the TXNA lesion is confined to a glycosylase found in a TXNs BGC. We also compared the cross-reactivity of the TxnU and LldU enzymes by testing the ability of TxnU4 to excise LLD adducts and of LldU1 to excise TXNA adducts, and found that both TxnU4 and LldU1 are capable of excising both TXNA and LLD adducts (Figure 6D), consistent with our results from HPLC analysis (Figure 4B and C).

Given the efficient activity of TxnU4 for TXNA lesions (Figure 4G), we were interested in determining whether TxnU and LldU could cleave other, less stable *N*7-alkyl-DNA adducts. We previously found that *E. coli* YcaQ readily excises 7mG (Figure 6E) and unhooks NM-ICLs generated from reaction of DNA with mechlorethamine (Figure 6F) (28,29). To our surprise, in contrast to YcaQ, neither TxnU4 nor LldU1 showed any significant activity toward 7mG (Figure 6E) or a NM-ICL (Figure 6G, H) after 30 min, despite the lower stability of these lesions relative to TXNs adducts. The inability of TxnU4 to act on these less stable *N*7-alkyl adducts and of other alkylpurine DNA glycosylases to process TXNA-DNA indicate that the TxnU/LldU enzymes are highly specific for their cognate natural products, and suggest that the enzymes likely recognize a specific feature of the TXNs-DNA substrates either directly through interaction with the compound or indirectly through the structural distortion to the DNA imposed by the intercalated adduct (Supplementary Figure S7).

### AP sites generated from TxnU4 cleavage of TXNA-DNA are inefficiently processed by EndoIV

**Figure 7.**
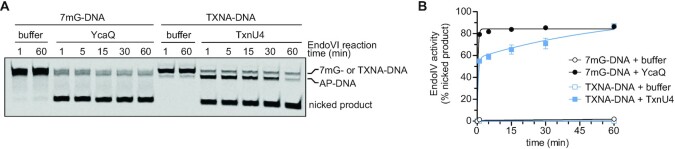
AP sites generated from TxnU4 action on TXNA-DNA are incised inefficiently by EndoIV. (**A**) Representative denaturing PAGE of EndoIV incision of AP-DNA generated from YcaQ hydrolysis of 7mG or TxnU4 hydrolysis of TXNA-G. 50 nM 7mG- or TXNA-DNA was incubated with either buffer or 5 nM YcaQ or TxnU4 for 2 h at 25°C to generate AP sites, followed by addition of EndoIV at a final concentration of 17 nM EndoIV and 40 nM DNA. EndoIV reactions were incubated at 37°C for the specified times prior to denaturing and electrophoresis. (**B**) Quantification of the gel in panel A. Data are mean ± SD (*n* = 3). 7mG data were fit to a one-phase exponential (*k* = 2.8 min^–1^, *R*^2^= 0.9975), and TXNA data were fit to a biphasic exponential (*k*_fast_ = 2.0 min^–1^, *k*_slow_ = 0.02 min^–1^, *R*^2^= 0.9755).

The AP site product of DNA glycosylase activity is a toxic intermediate of the BER pathway, and thus must be efficiently incised by an AP endonuclease for completion of the pathway. We therefore investigated the efficiency with which a bacterial AP endonuclease could act on the product of the TxnU4/TXNA-DNA reaction. When comparing various methods to cleave TxnU4-generated AP sites in our gel-based assay, we noticed that *E. coli* EndoIV did not fully incise the AP-DNA created by TxnU4 (Supplementary Figure S5A, B). The EndoIV reaction was carried out under the same conditions that show 100% incision activity from AP sites generated by AlkZ or YcaQ excision of 7mG (28,29), suggesting that the product of the TxnU4/TXNA-DNA reaction inhibits the AP endonuclease. We therefore followed up on this result by comparing the kinetics of EndoIV cleavage of AP sites generated by TxnU4/TXNA-DNA and YcaQ/7mG-DNA reactions (Figure 7). We wished to examine AP site processing without interference from residual glycosylase bound to either substrate or product DNA. Therefore, AP sites were generated under conditions that allow for completion of the glycosylase reaction with sub-saturating concentrations of protein with respect to DNA. We found that EndoIV incision of AP sites formed by YcaQ/7mG-DNA are rapidly and fully incised (*k*_obs_ = 2.8 min^–1^) within 5 min (Figure 7). In contrast, EndoIV incision of AP sites generated from TxnU4/TXNA-DNA showed biphasic kinetics. The first phase is consistent with the first enzymatic under our experimental conditions, and showed similar kinetics (*k*_fast_ = 2.0 min^–1^) as EndoIV activity on 7mG-produced AP sites. However, the second phase (i.e. subsequent turnovers) was 100-fold slower (*k*_slow_ = 0.02 min^–1^), suggesting that *E. coli* EndoIV is product inhibited when processing TXNA-generated AP sites. More importantly, the difference in EndoIV processing of TXNA and 7mG excision products indicates a difference in AP sites generated from the two lesions, the most likely rationale for which is that gutingimycin (TXNA-Gua) remains intercalated in the DNA after glycosylase excision. These data show that the AP-DNA/TXNA-Gua product poses a challenge for processing by *E. coli* IV, and suggests that a specialized AP endonuclease may be required for efficient BER of these lesions.

## DISCUSSION

In this study, HTH_42 superfamily proteins TxnU2/U4 and LldU1/U5 were discovered to provide cellular resistance to TXNA and LLD toxicity, respectively, providing an explanation for the evolutionary function of these proteins within the BGC of each antibiotic. Sequence (BLASTP) and structural (AlphaFold) analyses show that TxnU and LldU share homology with AlkZ and YcaQ (Supplementary Figure S6), and the *in vitro* enzymatic activity confirms that like AlkZ/YcaQ, both TxnU and LldU are monofunctional DNA glycosylases acting on *N*7-alkylguanine adducts (27–29). However, the TxnU/LldU enzymes differ from their HTH_42 homologs—and other alkylpurine DNA glycosylases—with respect to substrate specificity, catalytic machinery, and genomic context.

In terms of specificity, most alkylpurine DNA glycosylases hydrolyze 7mG in addition to their major substrates (23,28,29,40,54–56). Interestingly, despite the lower stability of the 7mG N-glycosidic bond, TxnU4 and LldU1 did not exhibit 7mG activity, indicating that TxnU/LldU specifically recognize TXNA-G and LLD-G as opposed to the instability in the *N*-glycosidic bond generated by substitution of guanine at N7 (1). Similarly, the TXNs-DNA lesions did not appear to be substrates for the other alkylpurine DNA glycosylases, including AlkZ/YcaQ and YtkR2/AlkD, which also act on bulky lesions (26,27,29,57,58). The lack of activity of TxnU/LldU for less stable *N*7-alkylguanine adducts and the inability of other glycosylases to hydrolyze TXNA-G indicate that TxnU and LldU are highly specific for their own natural products. The most significant differences between LLD/TXN-G and other known *N*7-alkylpurine glycosylase substrates are their ability to intercalate into the DNA base stack and their sugar substituents (Supplementary Figure S7). Based on the TXNA-DNA crystal structure, TXNA intercalates the d(GT/AC) base step and forms hydrogen bonds with the duplex DNA through the two sugar moieties, leading to the 4-sugar in the minor groove and the 13-sugar residing in the major groove (36). In addition, TXNA extrudes the base near the 3′ end of the alkylating site out of the helix, leading to an increased helical twist (36).

To our knowledge, TxnU and LldU are the only DNA glycosylases identified with activity toward intercalated DNA substrates. An AlkZ-derived homology model of TxnU4 docked against the TXNA-DNA crystal structure provides a rationale for this specificity (Supplementary Figure S7). Our previous work predicted that AlkZ employs two important secondary structural elements to engage the DNA substrate from opposite faces of the DNA—the β11/12-hairpin is posited to contact the lesion in the minor groove, and helix αI is predicted to make direct contact to the AZB compound from the major groove side (28,29) (Supplementary Figures S6,S7). Because the TXN compounds intercalate both strands of DNA, they protrude from both major and minor groove sides. Consequently, helix αI and the β11/12-hairpin likely contact TXNs from both grooves, with helix αI recognizing the C13- or C16-modified sugars on one end and the β11/12-hairpin recognizing C-4 modified sugar on the other end. Interestingly, the sequences and predicted structures of these two recognition elements are not conserved between AlkZ and TxnU/LldU (Supplementary Figures S6 and S7), consistent with their predicted roles in recognition of two different classes of natural products.

Regarding catalysis, the AlkZ/YcaQ/TxnU/LldU family of HTH_42 enzymes act on crosslinked or intercalated substates that are not likely to be extruded from the DNA, as observed for base-flipping glycosylases including human AAG and bacterial AlkA (19,23). Consistently, the HTH_42 enzymes, like their non-base-flipping counterparts YtkR2/AlkD, do not contain residues that would intercalate the DNA helix to stabilize an extruded nucleobase in the active site, nor do they contain a nucleobase binding pocket within the active site (57,59). Instead, the catalytic residues are pre-organized to contact the target N-glycosidic bond within an intact DNA duplex (28) (Supplementary Figures S6, S7). We previously showed that the catalytic motifs of the HTH_42 superfamily are divided into QΦQ and QΦD types (29). Sequence similarity network (SSN) analysis showed that the five proteins—AlkZ, LldU1/U5 and TxnU2/U4—are located in three different clades, in which TxnU2 and LldU5 are clustered into one clade, TxnU4 and LldU1 are clustered into another, and AlkZ clustered in a third (Figure 1D). The catalytic motif of TxnU2 and LldU5 is the same as AlkZ and belongs to the QΦQ type. However, the catalytic motifs of TxnU4 and LldU1 belong to neither QΦQ nor QΦD, but instead contain an HΦQ motif (Figure 5 and Supplementary Figure S6). Our structural models predict the HΦQ side chains to be in the same locations as those in AlkZ QΦQ, and thus either could reside close enough to the target TXNA-G or LLD-G nucleotide to catalyze hydrolysis (Supplementary Figures S6 and S7) (28). Interestingly, however, our mutational analysis revealed that HΦQ behaves differently than QΦQ and QΦD in two respects. First, mutation of only one residue affected base excision, in contrast to QΦQ (AlkZ) and YcaQ (QΦD), in which mutation of either residue within the motif affects base excision activity (28,29). Second, the two HΦQ motifs in TxnU4 and LldU1 have different effects for TXNA- and LLD-G adducts, respectively; the histidine in TxnU4 had the greater effect on excision of gutingimycin and the glutamine in LldU1 had the greater effect on LLD-G excision. The cross-reactivity of TxnU4 and LldU1 against TXNs and their high sequence similarity suggests that the two glycosylases have similar substrate recognition pockets, and thus the different effects of their His and Gln mutants most likely stem from the manner in which TXNA-G and LLD-G lesions are positioned within the active site (Supplementary Figure S7). These compounds are distinguished by the sugar substituents at position 13 (TXNA) and 16 (LLD) (Figure 1A), which reside in the major groove and thus likely are contacted by helix αI as described above (Supplementary Figure S7). Interestingly, TxnU4 and LldU1 contain a 10–15-amino acid insertion in helix αI that the AlphaFold model predicts forms a β-hairpin (Supplementary Figures S6 and S7). Steric interaction from this helix αI insertion with the unique 13- and 16-sugar substituents in the major groove would displace the TXNA- and LLD-DNAs differently, placing the target deoxyriboses of TXNA-G and LLD-G in proximity to His43 and Gln45, respectively (Supplementary Figure S7D). Thus, although TxnU4 and LldU1 share the same catalytic motif, the insertion in the predicted drug-binding αI-helix and the differences in sugar moieties in TXNs may alter how the two proteins engage their substrates. Consistent with this rationale, neither LldU5, AlkZ, nor YcaQ contain the αI helix insertion, and none of these show a preferential catalytic residue within QΦQ or QΦD motifs (28,29).

A growing number of specialized DNA glycosylases produced from the BGCs of genotoxic secondary metabolites have been determined, including those involved in self-resistance to AZB and yatakemycin/CC-1065 (26,27,60). Our cellular resistance/sensitivity assays demonstrate *txnU2/4* and *lldU1/5* are key determinants in self-resistance to TXNA/LLD. The presence of multiple copies of these DNA glycosylases is unique to the *txn* and *lld* BGCs, and may provide redundancy to ensure repair of the highly genotoxic TXN metabolites, in contrast to the lethality of AlkZ knockouts in azinomycin B-producing *S. sahachiroi* (27). Based on our finding that TxnU4 and LldU1 play the major roles in toxin resistance, it is interesting to speculate that TxnU2 and LldU5 play more secondary roles, such as removing lesions formed by TXN derivatives generated from catabolism of TXNA/LLD.

The subsequent BER steps necessary for repair of DNA lesions generated from secondary metabolites, and the roles of other pathways (e.g., NER) are remaining questions. Regarding BER, our finding that *E. coli* EndoIV processed TXNs AP-sites less efficiently than 7mG-derived AP-sites suggests that specialized nucleases act on the AP-DNA/TXN-Gua product, as predicted for the putative *ytkR4* and *ytkR5* nucleases located within yatakemycin BGC (26,58,61). Although there do not appear to be any nucleases within the *txn*/*lld* clusters, genomic analysis reveals both ExoIII and EndoIV orthologs in TXNA/LLD producing strains (and two ExoIII paralogs in the case of *S. bottropensis*). Given the bulky, helix-distorting nature of these compounds, it is also likely that NER or other pathways play a role in their repair, as previously shown for yatakemycin-family and NM-ICL-DNA lesions (29,58,62–64). Indeed, *S. vinaceusdrappus* and *S*. *bottropensis* contain one and three UvrA paralogs, respectively. It is also possible that TXN-DNA lesions are recognized by other enzymes outside of BER or NER, as reported for the structure-specific AziN nuclease within the AZB BGC (65). More work is needed to elucidate the full landscape of cellular mechanisms of repair of these unique DNA damaging agents. Taken together, this work characterizes a unique family of DNA glycosylases from the HTH_42 superfamily that act on heavily functionalized, intercalated DNA adducts, and provides further evidence for that DNA glycosylases residing in BGCs have evolved an exquisite specificity for aberrant nucleotides formed by their cognate genotoxic natural products.

## DATA AVAILABILITY

Strains and plasmids used in this study are available from the authors upon request. Sequences of *txn*and *lld*BGCs are available in GenBank under accession no. KP410250 and MK501817.

## Supplementary Material

gkac085_Supplemental_File
