# Supplementary material for: Base excision repair system targeting DNA adducts of trioxacarcin/LL-D49194 antibiotics for self-resistance
Source: Nucleic Acids Res. 2022 Feb 22;50(5):2417–30. doi: 10.1093/nar/gkac085 (PMC8934636; doi:10.1093/nar/gkac085)
Supplement: gkac085_Supplemental_File [file gkac085_supplemental_file.pdf]

Supplementary Information for

Base excision repair system targeting DNA adducts of trioxacarcin/LL-D49194 antibiotics for self-resistance

Xiaorong Chen[1,2,†], Noah P. Bradley[3,†], Wei Lu[2,†], Katherine L. Wahl[3], Mei Zhang[2], Hua Yuan[2], Xian-Feng Hou[2], Brandt F. Eichman[3,4,*], and Gong-Li Tang[1,2,*]

[1] School of Chemistry and Materials Science, Hangzhou Institute for Advanced Study, University of Chinese Academy of Sciences, 1 Sub-lane Xiangshan, Hangzhou 310024, China.
[2] State Key Laboratory of Bioorganic and Natural Products Chemistry Center for Excellence in Molecular Synthesis, Shanghai Institute of Organic Chemistry, University of Chinese Academy of Sciences, Chinese Academy of Sciences, Shanghai 200032, China.
[3] Department of Biological Sciences, Vanderbilt University, Nashville, TN 37232.
[4] Department of Biochemistry, Vanderbilt University, Nashville, TN 37232.

[†] These authors contributed equally to this work.

* To whom correspondence should be addressed. Tel: +86-21-54925113; Fax: +86-21-64166128; Email: gltang@sioc.ac.cn
Correspondence may also be addressed to Brandt F. Eichman. Tel: +1 615 936 5233; Fax: +1 615 936 2211; Email: brandt.eichman@vanderbilt.edu

<u>Table of contents</u>

Supplementary Materials and Methods
- Gene deletion of *txnU2*/*txnU4* and *lldU1*/*lldU5*
- Structural Modeling

Supplementary Tables
- Table S1. Strains used in this study
- Table S2. Oligodeoxynucleotides used in this study

Supplementary Figures
- Figure S1. PCR confirmation of the mutants of *S. vinaceusdrappus* and *S. bottropensis*
- Figure S2. LC-MS analysis of the cleavage ability of enzymes TxnU2, TxnU4, LldU1 and LldU5 on alkylated adducts TXNA-DNA (A) and LLD-DNA (B)
- Figure S3. Tandem MS/MS spectrum analysis of enzymatic product gutingimycin (A) and LLD-Gua (B)
- Figure S4. Extended time course analysis of TxnU4 on TXNA-G adducts
- Figure S5. Controls and raw data for gel-based base excision assays
- Figure S6. Conservation of TxnU/LldU and AlkZ enzymes
- Figure S7. DNA binding models of AlkZ and TxnU4

Supplementary references

Supplementary Materials and Methods

Gene deletion of *txnU2*/*txnU4* and *lldU1/lldU5*

The gene interruption mutant strains of *txnU2* and *txnU4* were constructed with REDIRECT Technology as previously described (1,2). The homologous fragment of each target gene containing an *acc(3)IV* (apramycin resistance gene) and *oriT* was amplified from the plasmid pIJ773 using primer tg-txnU2-F/tg-txnU2-R and tg-txnU4-F/tg-txnU4-R, respectively (Table S2). The PCR template was then electro-transformed into the fosmid pTG5001 to generate the mutant fosmids pTGtxnU2 and pTGtxnU4. These mutant fosmids were introduced respectively into the *Streptomyces bottropensis* NRRL 12051 using *E. coli-Streptomyces* intergeneric conjugation. The deletion mutants Δ*txnU2* and Δ*txnU4* were screened out as previously published (1,2) and respectively confirmed by PCR using the primers txnU2-yz-F/ txnU2-yz-R, txnU4-yz-F/ txnU4-yz-R (Table S2).

The in-frame deletion mutants of gene *lldU1* and *lldU5* were constructed by using the temperature-sensitive plasmid pKC1139. The left and the right homologous fragments of each gene were amplified from the genome of *S. vinaceusdrappus* NRRL 15735 with primers lldU1-pKC1139-L-For/ lldU1-pKC1139-L-Rev, lldU1-pKC1139-R-For/ lldU1-pKC1139-R-Rev, lldU5-pKC1139-L-For/ lldU5-pKC1139-L-Rev and lldU5-pKC1139-R-For/ lldU5-pKC1139-R-Rev, respectively. The two homologous fragments of each gene were subsequently cloned into the vector pKC1139 using enzyme ligation to produce the deletion plasmid pKC-lldU1 and pKC-lldU5. The deletion plasmids were introduced into the *S. vinaceusdrappus* NRRL 15735 using *E. coli-Streptomyces* intergeneric conjugation. The in-frame deletion mutants Δ*lldU1* and Δ*lldU5* were screened out and confirmed by PCR using the primers lldU1-yz-F/ lldU1-yz-R, lldU5-yz-F/ lldU5-yz-R (Table S2).

Structural Modeling

The model of TxnU4 bound to TXNA-DNA was based on our previous model of AlkZ docked against an AZB-ICL-DNA (3). A TxnU4 homology model calculated using AlphaFold2 (4) was superimposed onto AlkZ. The crystal structure of TXNA-DNA was superimposed onto the AZB-ICL-DNA model using the adducted guanine as an anchor point. Protein and DNA were treated as rigid bodies, and no energy minimization was performed. All modeling was performed in PyMOL v2.3.2 (Schrödinger, Inc.).

Table S1. Strains used in this study.

**Strain or plasmid**	**Characteristics**	**Source or reference**
Streptomyces bottropensis	Trioxacarcin A producing strain (wild type)	NRRL 12051
Δ*txnU2*	In-frame deletion mutant of *txnU2*	This study
Δ*txnU4*	In-frame deletion mutant of *txnU4*	This study
S. vinaceusdrappus	LL-D49194α1and LL-D49194β2 producing strain (wild type)	NRRL 15735
Δ*lldU1*	In-frame deletion mutant of *lldU1*	This study
Δ*lldU2*	In-frame deletion mutant of *lldU5*	This study
S. lividans 1326	Trioxacarcin A, LL-D49194α1and LL-D49194β2 sensitive strain, used for the heterologous expression of the genes *txnU2*, *txnU4*, *lldU1* and *lldU5*	
S. lividans::pSET152	Heterologous expression strain with empty vector, served as negative control strain	This study
S. lividans::*txnU2*	Heterologous expression strain with *txnU2* gene	This study
S. lividans::*txnU4*	Heterologous expression strain with *txnU4* gene	This study
S. lividans::*lldU1*	Heterologous expression strain with *lldU1* gene	This study
S. lividans::*lldU5*	Heterologous expression strain with *lldU5* gene	This study
E. coli BL21 (DE3)	Host strain for Protein overexpression	Invitrogen
pET28a/E. coli BL21	Protein overexpression strain of with empty vector, served as negative control strain	This study
txnU2-pET28a/*E. coli* BL21	Protein overexpression strain of TxnU2	This study
txnU4-pET28a/*E. coli* BL21	Protein overexpression strain of TxnU4	This study
lldU1-pET28a/*E. coli* BL21	Protein overexpression strain of LldU1	This study
lldU5-pET28a/*E. coli* BL21	Protein overexpression strain of LldU5	This study
plasmid		
pTG5001	Fosmid vector containing TXN gene cluster	This study
pET28a	Protein expression vector with His_6 tag	Novagen
pBG102	Protein expression vector with N-terminal His_6-SUMO tag	This study
pIJ773	Vector containing the apramycin resistance gene *aac(3)IV* and *oriT*	This study
pTGtxnU2	pTG5001 derivative for gene deletion of *txnU2*	This study
pTGtxnU4	pTG5001 derivative for gene deletion of *txnU4*	This study
pKC1139	Streptomyces temperature-sensitive plasmid for gene deletion, Apr^r	(5)
pKC-lldU1	pKC1139 derivative for gene deletion of *lldU1*	This study
pKC-lldU1	pKC1139 derivative for gene deletion of *lldU5*	This study

Table S2. Oligodeoxynucleotides used in this study.

Oligo Name	Sequence (5′→3′)[b]	Use
tg-txnU2-F	GGCTGCGGACAAGGGTTCGAGGGGCTCAAC CGTCACCTGATTCCGGGGATCCGTCGACC	*txnU2* PCR targeting primer
tg-txnU2-R	ACCTACACCAATCCGCTGCGCTACCTGAACG GCGTGCGCTGTAGGCTGGAGCTGCTTC	*txnU2* PCR targeting primer
tg-txnU4-F	GGCTGCGGACAAGGGTTCGAGGGGCTCAAC CGTCACCTGATTCCGGGGATCCGTCGACC	*txnU4* PCR targeting primer
tg-txnU4-R	ACCTACACCAATCCGCTGCGCTACCTGAACG GCGTGCGCTGTAGGCTGGAGCTGCTTC	*txnU4* PCR targeting primer
txnU2-yz-F	AACCGGTCCTTGCTGCAG	For confirmation of *txnU2* deletion
txnU2-yz-R	GGCCGCGAGATCTGCCTT	For confirmation of *txnU2* deletion
txnU4-yz-F	GGCGCAGACTGCTGTCCTGT	For confirmation of *txnU4* deletion
txnU4-yz-R	TTACGGGGTCCTGACACTGAT	For confirmation of *txnU4* deletion
lldU1-pKC1139-L-For	AAAAAGCTTGTGTCCTGGCACGAACGAC	Primer of *lldU1* left homologous fragments
lldU1-pKC1139-L-Rev	AAATCTAGAGCCGTGCGTCTTCACCAG	Primer of *lldU1* left homologous fragments
lldU1-pKC1139-R-For	AAATCTAGACTCACGAAACTCTCCACGGC	Primer of *lldU1* right homologous fragments
lldU1-pKC1139-R-Rev	AAAGAATTCCGAGCAAGGTCAGCGGAT	Primer of *lldU1* right homologous fragments
lldU5-pKC1139-L-For	AAAAAGCTTGGCGTAGCCGACGAAACT	Primer of *lldU5* left homologous fragments
lldU5-pKC1139-L-Rev	AAATCTAGAATGTCGAGGACCGACACG	Primer of *lldU5* left homologous fragments
lldU5-pKC1139-R-For	AAATCTAGACTGGGTGGTGCGCAAATG	Primer of *lldU5* right homologous fragments
lldU5-pKC1139-R-Rev	AAAGAATTCGACCGGCTCCGTGATACG	Primer of *lldU5* right homologous fragments
lldU1-yz-F	CGCCAAGCACGAAGGAGA	For confirmation of *lldU1* deletion
lldU1-yz-R	GCCGTACGCCAGTGACC	For confirmation of *lldU1* deletion
lldU5-yz-F	GGCACATGAAGCGGGGG	For confirmation of *lldU5* deletion
lldU5-yz-R	AACGGTTCTCGGTCCTGTG	For confirmation of *lldU5* deletion
U1_H43A_FP	GTGCGGCGCGGCGGCGCAGGTTCTG	Mutational primer
U1_H43A_RP	AGCGCACGCACCAGTTCC	Mutational primer
U1_Q45A_FP	CGCGCACGCGGCGGTTCTGAGCGCG	Mutational primer
U1_Q45A_RP	CCGCACAGCGCACGCACC	Mutational primer
U4_H43A_FP	GTGCGGTGCGGCGGCGCAAGTTC	Mutational primer
U4_H43A_RP	ATCGCACGCACAATTTCC	Mutational primer
U4_Q45A_FP	TGCGCATGCGGCGGTTCTGAGCGCG	Mutational primer
U4_Q45A_RP	CCGCACATCGCACGCACA	Mutational primer
U5_Q41A_FP	GGTTGCGATGGCGGGTCAAGAACCG	Mutational primer
U5_Q41A_RP	AGGTGCTCAACCACACGC	Mutational primer
U5_Q43A_FP	GATGCAGGGTGCGGAACCGGATGCG	Mutational primer

U5_Q43A_RP	GCAACCAGGTGCTCAAC	Mutational primer
TXN/LLD_HPLC	AACCGGTT	Base excision, Fig. 4B-C, Fig. S2
7mG_Top [c]	Cy5-CACCACTACACC(7mG)ATTCCTTACAAC	Base excision, Fig. 5E, Fig. S5E
7mG_Bottom	GTTGTAAGGAATCGGTGTAGTGGTG	Base excision, Fig. 5E, Fig. S5E
NM_Top	FAM-AAAAATAAAAGTCAAATAAAAATAAA	Base excision, Fig. 5H
NM_Bottom	Cy5-TTTATTTTTATTTGACTTTTATTTTT	Base excision, Fig. 5H
TXN/LLD_Top	Cy5-ATCGAATCCAATGTCTAAGGAATTCT	Base excision, Fig. 4E-H, 5A-D, 6, 7, Fig. S5
TXN/LLD_Bottom	AGAATTCCTTAGACATTGGATTCGAT	Base excision, Fig. 4E-H, 5A-D, 6, 7, Fig. S5

[a] Oligodeoxynucleotides were dissolved in TE buffer (10 mM Tris•HCl pH 8.0, 1 mM EDTA) and stored at -20°C. Cy5/FAM oligonucleotides were stored in the dark.
[b] The underlined G is the site of alkylation. FAM, 6-carboxyfluorescein; Cy5, cyanine 5.
[c] The blue text in 7mG_Top denotes nucleotides that are incorporated enzymatically

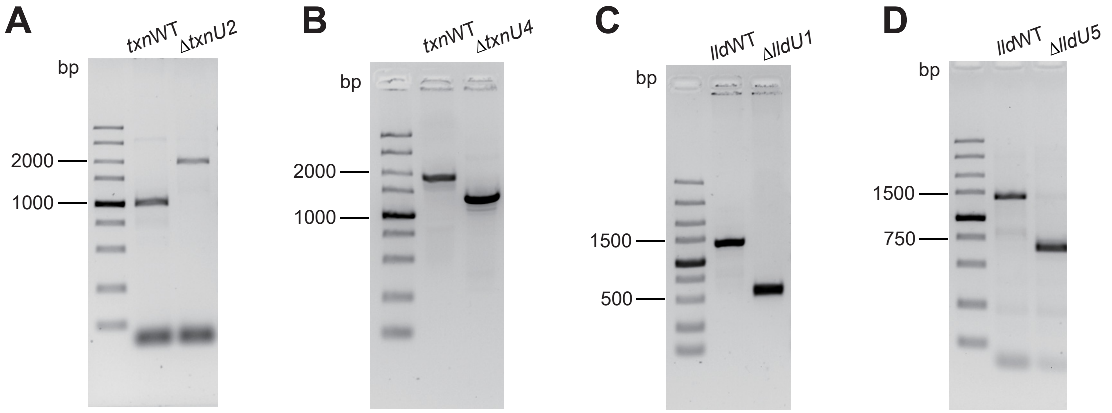

Figure S1. PCR confirmation of the mutants of *S. vinaceusdrappus* and *S. bottropensis*. (**A**) PCR verification of *txnU2* deletion mutant strain Δ*txnU2*. A 2000-bp DNA fragment was obtained from Δ*txnU2* with using primers txnU2-yz-F/ txnU2-yz-R, while a 1000-bp fragment was amplified from wild type strain *S. vinaceusdrappus* (txnWT). (**B**) PCR confirmation of *txnU4* deletion mutant strain Δ*txnU4*. A 1200-bp DNA fragment was obtained from Δ*txnU4* by using primers txnU4-yz-F/ txnU4-yz-R, while a 1900-bp fragment was amplified from wild type strain *S. vinaceusdrappus* (txnWT). (**C**) PCR confirmation of *lldU1* deletion mutant strain Δ*lldU1*. A 1400-bp DNA fragment was obtained from Δ*lldU1* by using primers lldU1-yz-F/ lldU1-yz-R, while a 560-bp fragment was amplified from wild type strain *S. bottropensis* (lldWT). (**D**) PCR confirmation of *lldU5* deletion mutant strain Δ*lldU5*. A 1450-bp DNA fragment was obtained from Δ*lldU5* by using primers lldU5-yz-F/ lldU5-yz-R, while a 630-bp fragment was amplified from wild type strain *S. bottropensis* (lldWT).

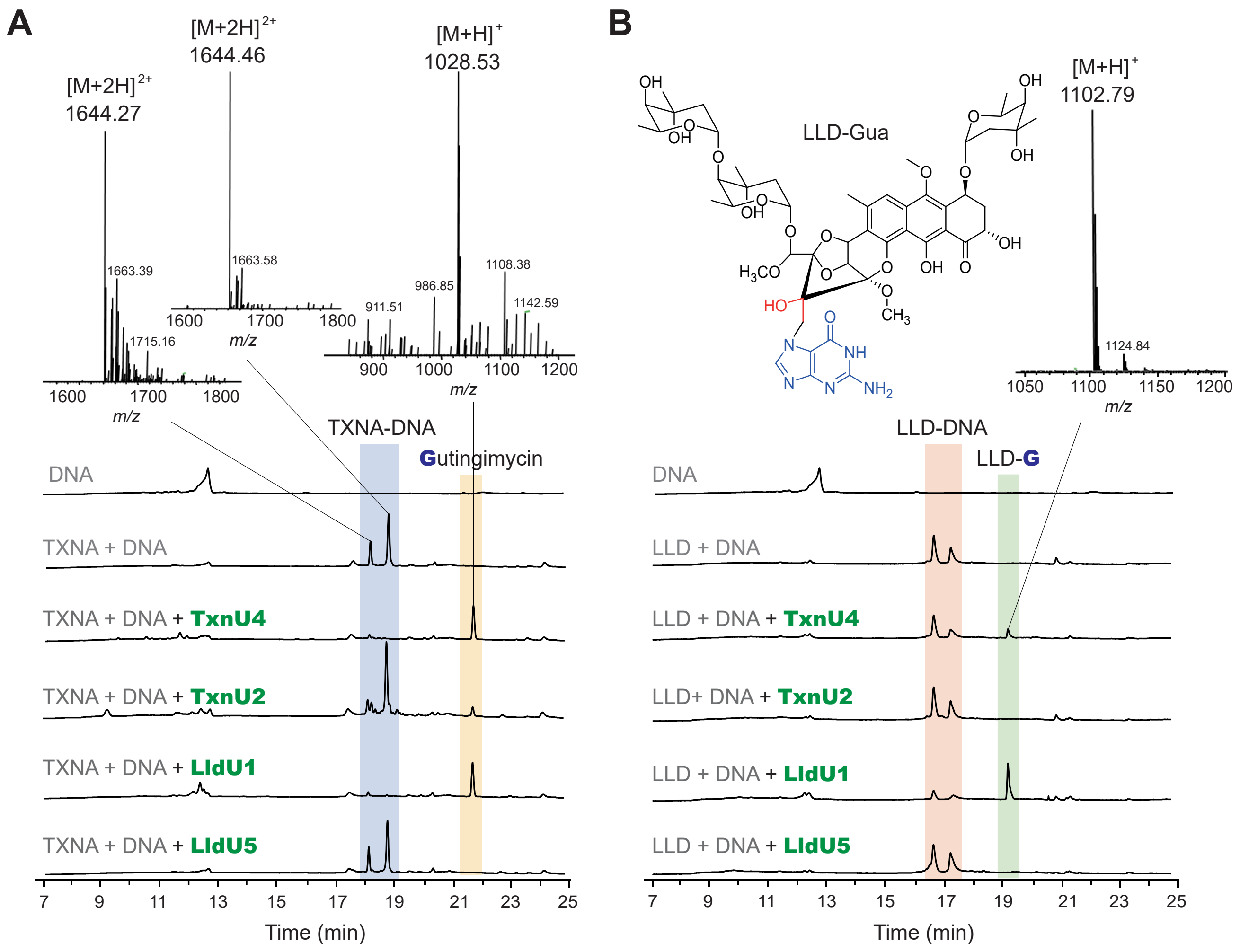

Figure S2. LC-MS analysis of the cleavage ability of enzymes TxnU2, TxnU4, LldU1 and LldU5 on alkylated adducts TXNA-DNA (A) and LLD-DNA (B). An 8-bp oligodeoxynucleotide duplex d(AACCGGTT) was pre-incubated with TXNA or LLD at 16°C for 2 hr, followed by treatment with enzymes TxnU2, TxnU4, LldU1 and LldU5. The reaction mixtures were analyzed by LC-MS at 271 nm absorbance. (**A**) After treatment with TXNA, two new peaks appeared at 18.2 and 18.9 min. The *m/z* of the both two peaks were 1644, which was consistent with that of the $[M+2H]^{2+}$ ion of the monoalkylated adduct generated by covalent binding of one molecule TXNA to the guanine within the duplex d(AACCGGTT). The *m/z* of product (gutingimycin) excised by the enzymes was 1028.53. (**B**) With the addition of TxnU4 and LldU1, a new peak (LLD-Gua) with m/z 1028 appeared.

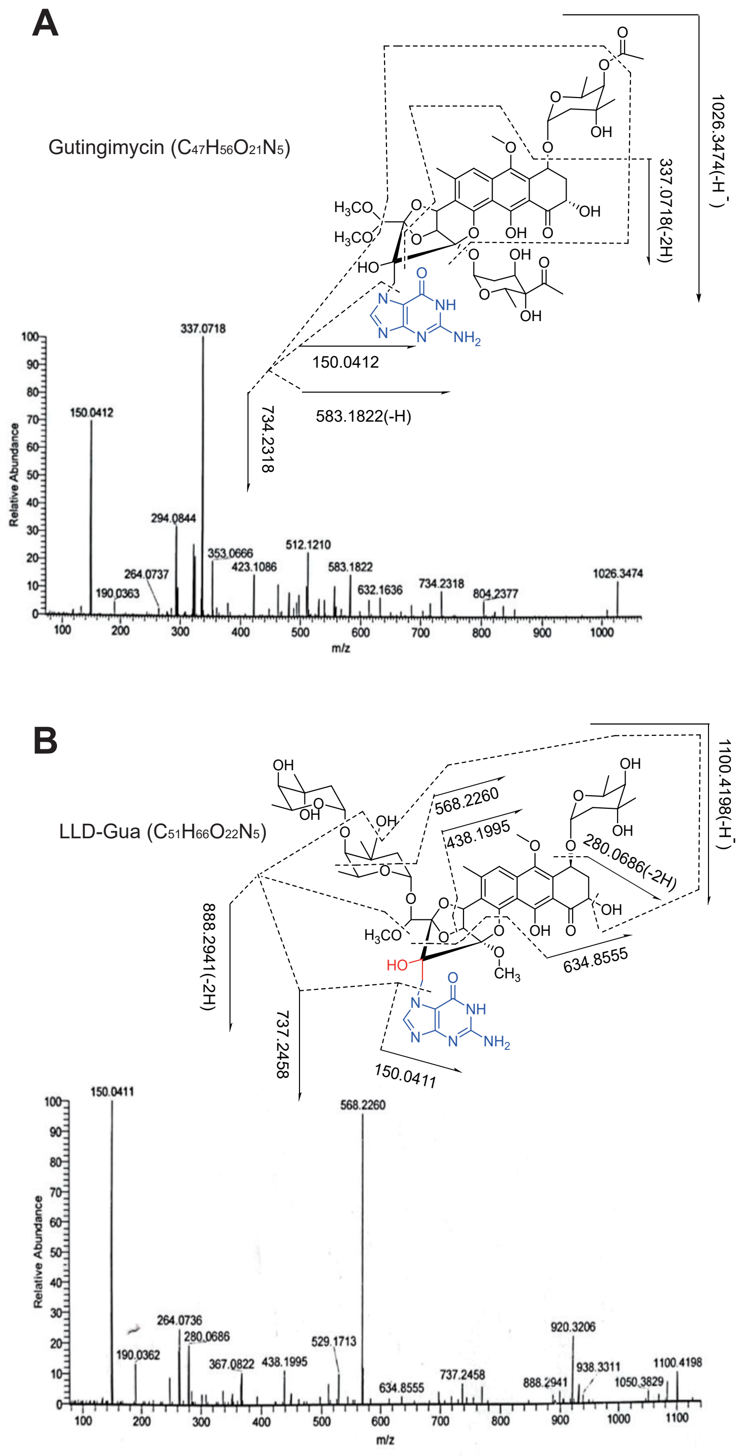

Figure S3. Tandem MS/MS spectrum analysis of enzymatic product gutingimycin (A) and LLD-Gua (B). The tandem MS/MS spectrum of gutingimycin derived from the HRESIMS *m/z* 1026.3474 ($[M-H]^-$, calcd. for $C_{47}H_{57}O_{21}N_5$, 1026.3546). The tandem MS/MS spectrum of LLD-Gua derived from the HRESIMS *m/z* 1100.4198 ($[M-H]^-$, calcd. for $C_{51}H_{68}O_{22}N_5$, 1100.4298).

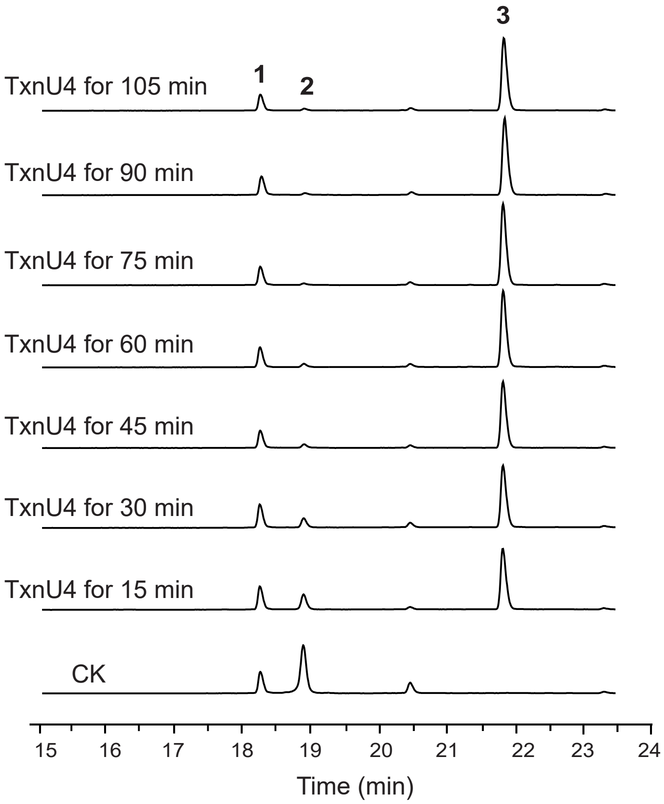

1: 5′-AACC(TXNA-G)GTT-3′

2: 5′-AACCG(TXNA-G)TT-3′

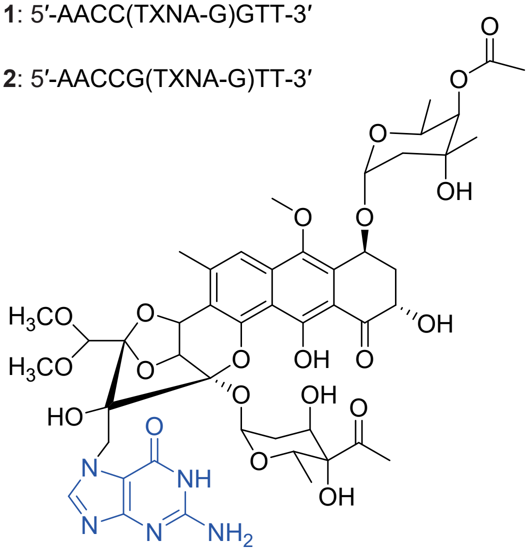

3: Gutingimycin

Figure S4. Extended time course analysis of TxnU4 on TXNA-G adducts. Oligodeoxynucleotide duplex d(AACCGGTT) was pre-incubated with TXNA at 16°C for 2 hr, followed by treatment with or without 2 μM TxnU4 for different times (15, 30, 45, 60, 75, 90, 105 min). The reaction mixtures were analyzed by LC-MS at 400 nm absorbance. CK: the TXNA alkylation products treated without TxnU4. 1: alkylation product 5′-AACC(TXNA-G)GTT-3′. 2: alkylation product 5′-AACCG(TXNA-G)TT-3′. 3: cleavage product gutingimycin

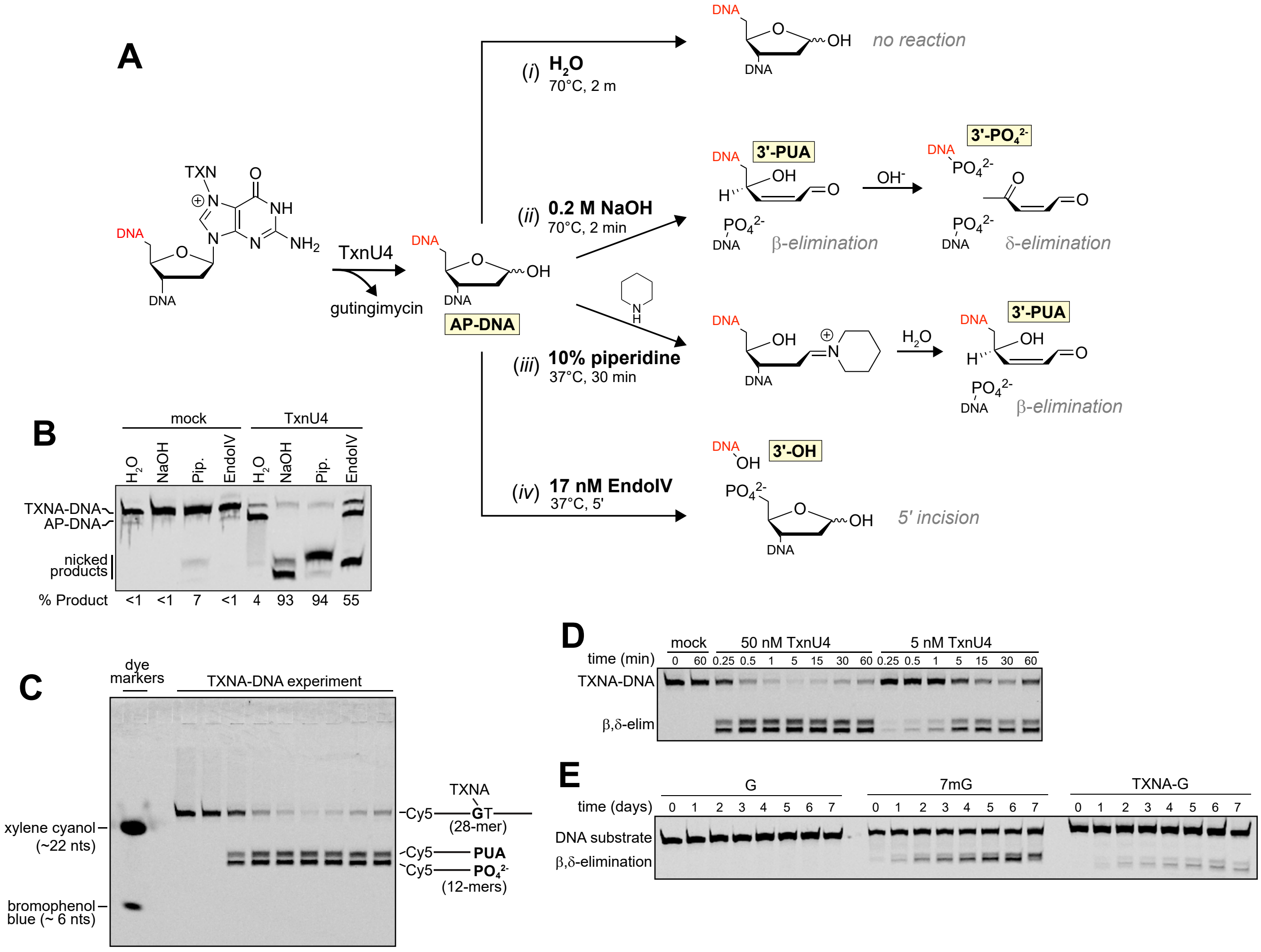

Figure S5. Controls and raw data for gel-based base excision assays. (**A**,**B**) Comparison of methods to cleave glycosylase-generated abasic sites. (**A**) Schematic of glycosylase reactions and subsequent AP site work-up by four methods (i-iv). (i) AP sites generated by TxnU4 activity on TXNA adducts can be incubated with H_2O to give no reaction. (ii) AP sites can be cleaved with NaOH to generate a 3′-PUA (3′-phospho-α,β-unsaturated aldehyde) through β-elimination, as well as a 3′-PO_4^{2-} species via δ-elimination. (iii) Piperidine treatment of AP sites proceeds through a Schiff base imine intermediate which undergoes β-elimination creating a 3′-PUA. Piperidine treatment at 37°C for 30 min is not sufficient to catalyze δ-elimination. (iv) Treatment of AP-DNA with bacterial EndoIV results in the incision of the DNA 5′ to the abasic site, leading to a 3′-OH at the 3′-terminus. (**B**) Denaturing PAGE showing results from the four AP site work-ups shown in panel A. 50 nM Cy5-TXNA-DNA was incubated with either 5 nM TxnU4 or no enzyme (mock) in glycosylase buffer for 2 hr at 25°C. Reactions were quenched by four different methods: H_2O, NaOH, piperidine, and EndoIV. For H_2O and NaOH workups, 1 μL of H_2O or 1 M NaOH (0.2 M final concentration) was added to a 4-μL reaction aliquot and heated at 70°C for 2 min. For the piperidine reaction, 1 μL of 50% piperidine (10% v/v final concentration) was added to a 4-μL reaction aliquot and heated at 37°C for 30 min. EndoIV incision reactions were performed by adding 1 μL of 83 nM EndoIV (17 nM final concentration) to a 4-μL reaction aliquot and heating at 37°C for 5 min. All reactions were subsequently denatured at 70°C for 5 min in 5 μL formamide/blue dextran loading buffer and electrophoresed on a 20% polyacrylamide/8 M urea gel. (**C**) Relative gel migration of substrate and product DNA. Shown is a 20% polyacrylamide / 8M urea gel containing a Cy5-TXNA-DNA (28mer) and β/δ-elimination products (12-mers) generated from reaction with TxnU4 followed by hydroxide treatment. The gel was imaged for Cy5 fluorescence (633 nm excitation, 670 nm emission). The far-left lane contains a mixture of fluorescent tracking dyes, xylene cyanol and bromophenol blue, which migrate at approximately 22 and 6 nts, respectively, in a 20% denaturing PAGE gel (6). (**D**) Representative denaturing PAGE for kinetic data shown in Figure 4G. (**E**) Representative gel from which Figure 6B data were quantified.

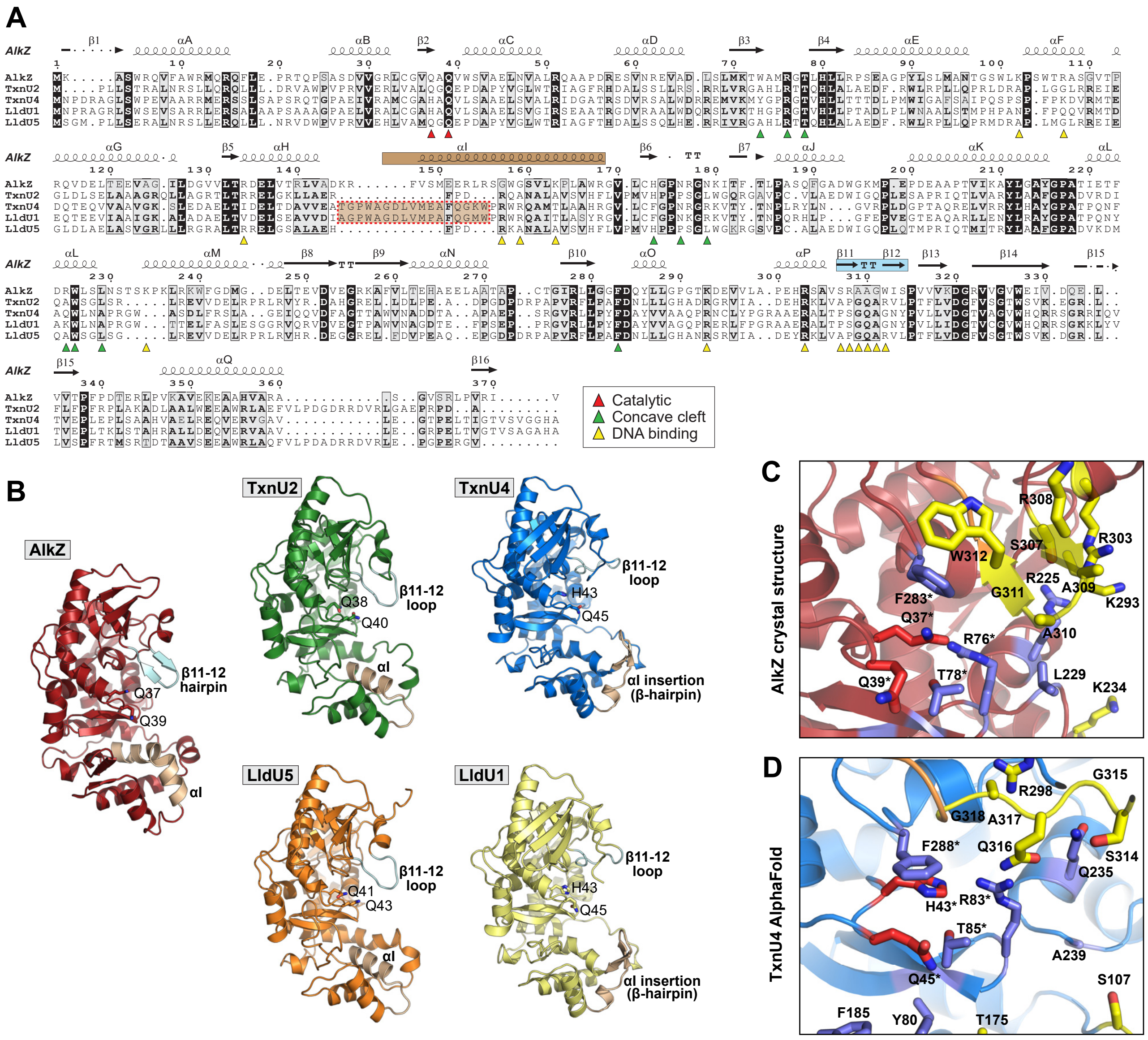

Figure S6. Conservation of TxnU/LldU and AlkZ enzymes. (**A**) Sequence alignment of AlkZ, TxnU2/U4, and LldU1/U5 generated using T-Coffee Expresso (7) and ESPript 3.0 (8). Secondary structure above the sequences is derived from the AlkZ crystal structure (PDB ID 5UUJ) (3). Helix αI and the β11/12-hairpin predicted to contact the DNA lesion are colored brown and cyan, respectively. The dashed red box highlights the helix αI insertion unique to TxnU4 and LldU1. Triangles below the sequences indicate catalytic (red) or DNA binding (yellow) residues, and residues residing within the concave cleft (green). (**B**) Comparison of AlkZ crystal structure (PDB ID 5UUJ) to TxnU/LldU models generated by AlphaFold2 (4). Catalytic QΦQ and HΦQ residues are in ball-and-stick, and putative lesion-binding secondary structural elements are highlighted brown and cyan as in panel A. (**C**,**D**) Active sites of AlkZ (**C**) and TxnU4 (**D**). Catalytic residues are red, β11-12-loop residues are yellow, and residues protruding into the DNA binding channel are blue.

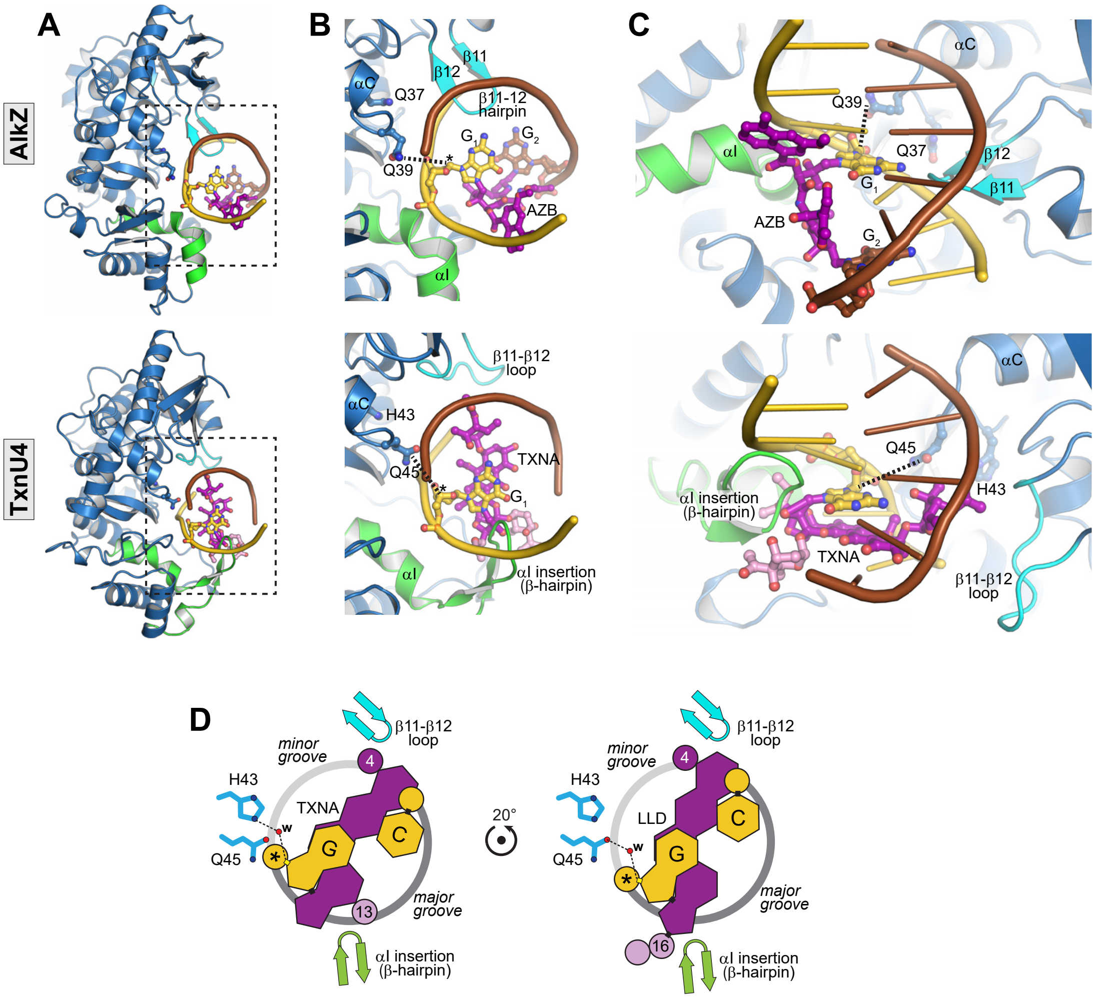

Figure S7. DNA binding models of AlkZ and TxnU4. The AlkZ crystal structure (PDB ID 5UUJ) docked against an AZB-ICL-DNA model (9) is on top and the TxnU4 AlphaFold2 model docked against the TXNA-DNA crystal structure (10) is on bottom. (**A**) DNA is docked into the concave cleft containing active site residues (ball-and-stick) and secondary structural elements (helix αI, green; β11-12 hairpin, cyan) predicted to contact the DNA lesion (3). (**B**) Zoom-in of region outlined by the dashed box in panel A. AZB and TXNA are colored purple. Sugar substituents on TXNA that differ between LLD are colored pink. Guanine residues covalently attached to the natural products are shown in ball-and-stick and C1′ of the adducted nucleotide is labeled with an asterisk. Dashed lines represent putative water-mediated contacts between the C-terminal glutamine of the QΦQ and HΦQ catalytic motifs and the target C1′. In both models, slight rotation of the DNA around the helical axis would place the adducted nucleotide in proximity to Q37 (AlkZ) or H43 (TxnU4). (**C**) Side view looking into the concave cleft. Helix αI (green) protrudes into the major groove side of the lesion and is predicted to interact with AZB and TXNA directly. The β11-12 hairpin in AlkZ and corresponding loop in the TxnU4 model are positioned on the minor groove side of the lesion. Because TXNA (and LLD) intercalate the DNA and span both major and minor grooves, the β11-12 loop likely interacts with the intercalated compounds directly. (**D**) Schematic of TxnU4/TXNA-DNA (left) and LldU1/LLD-DNA (right) complexes, viewed down the helical axis and colored as in panels A-C. The grey circle represents the DNA backbone, with major and minor grooves colored dark and light grey, respectively. Sugar moieties are depicted as circles and labeled numerically with their position in TXN. Unique sugar substituents between TXNA and LLD are pink. The catalytic water is labeled "w". TXNA-DNA is rotated 20° clockwise relative to LLD-DNA, which places the target N-glycosidic bond (yellow rectangle) within water-mediated hydrogen bonding distance to H43. The DNA in each complex is likely held in place by interactions between helix αI insertion and the unique sugar substituents on TXNA and LLD.

Supplementary references

1. Gust, B., Challis, G.L., Fowler, K., Kieser, T. and Chater, K.F. (2003) PCR-targeted *Streptomyces* gene replacement identifies a protein domain needed for biosynthesis of the sesquiterpene soil odor geosmin. *Proceedings of the National Academy of Sciences of the United States of America*, **100**, 1541-1546.
2. Zhang, M., Hou, X.F., Qi, L.H., Yin, Y., Li, Q., Pan, H.X., Chen, X.Y. and Tang, G.L. (2015) Biosynthesis of trioxacarcin revealing a different starter unit and complex tailoring steps for type II polyketide synthase. *Chemical science*, **6**, 3440-3447.
3. Mullins, E.A., Warren, G.M., Bradley, N.P. and Eichman, B.F. (2017) Structure of a DNA glycosylase that unhooks interstrand cross-links. *Proceedings of the National Academy of Sciences of the United States of America*, **114**, 4400-4405.
4. Jumper, J., Evans, R., Pritzel, A., Green, T., Figurnov, M., Ronneberger, O., Tunyasuvunakool, K., Bates, R., Žídek, A., Potapenko, A. *et al.* (2021) Highly accurate protein structure prediction with AlphaFold. *Nature*, **596**, 583-589.
5. Hopwood, D.A., Kieser, T., Bibb, M.J., Buttner, M.J. and Chater, K.F. (2000) Practical Streptomyces Genetics. *John Innes Foundation*.
6. Sambrook, J. and Russell, D.W. (2001) Gene Cloning. A Laboratory Manual, 3rd Ed. *Cold Spring Harbor Laboratory Press*.
7. Notredame, C., Higgins, D.G. and Heringa, J. (2000) T-Coffee: A novel method for fast and accurate multiple sequence alignment. *J Mol Biol*, **302**, 205-217.
8. Robert, X. and Gouet, P. (2014) Deciphering key features in protein structures with the new ENDscript server. *Nucleic Acids Res*, **42**, W320-324.
9. Alcaro, S. and Coleman, R.S. (2000) A molecular model for DNA cross-linking by the antitumor agent azinomycin B. *J Med Chem*, **43**, 2783-2788.
10. Pfoh, R., Laatsch, H. and Sheldrick, G.M. (2008) Crystal structure of trioxacarcin A covalently bound to DNA. *Nucleic acids research*, **36**, 3508-3514.
